# Geometry-Driven Structural Efficiency and Normative Performance of Miriti-Based Sandwich Composite Roofing Tiles

**DOI:** 10.3390/polym18080907

**Published:** 2026-04-08

**Authors:** Ana Célia Sousa da Silva, Maurício Maia Ribeiro, Douglas Santos Silva, Raí Felipe Pereira Junio, Sergio Neves Monteiro, Jean da Silva Rodrigues

**Affiliations:** 1 Materials Engineering Program, Federal Institute of Education, Science and Technology of Pará—IFPA, Avenida Almirante Barroso, 1155, Marco, Belém 66093-020, PA, Brazil; ac.sousa999@gmail.com (A.C.S.d.S.); jean.rodrigues@ifpa.edu.br (J.d.S.R.); 2 Federal Institute of Education, Science and Technology of Pará—IFPA, Estrada do Icuí Guajará, Ananindeua 67125-000, PA, Brazil; mauricio.maia@ifpa.edu.br; 3 Department of Materials Science, Military Institute of Engineering—IME, Praça General Tibúrcio, 80, Praia Vermelha, Urca, Rio de Janeiro 22290-270, RJ, Brazil; raivsjfelipe@ime.eb.br (R.F.P.J.); sergio.neves@ime.eb.br (S.N.M.)

**Keywords:** sandwich composites, lignocellulosic core, miriti wood, composite roofing tiles, stiffness-to-weight efficiency

## Abstract

This work experimentally evaluates the geometry-driven structural efficiency and normative performance of sandwich-type composite roofing tiles composed of a miriti wood core and fiberglass-reinforced polymer faces. Trapezoidal-profile tiles were manufactured by hand lay-up and assessed according to ABNT NBR 16753, including visual inspection, fiber content, water absorption, apparent flexural behavior, deformation resistance, and impact resistance. The miriti core exhibited an extremely low mean density of 0.091 ± 0.008 g/cm^3^ (CV ≈ 8.8%), enabling lightweight sandwich configurations with an average overall thickness of approximately 8 mm. Fiberglass contents ranged from 27.5% to 32.1% by mass. Sealed sandwich specimens showed median water uptake values of approximately 2.5% after 2 h and 6.0% after 24 h immersion. Deformation resistance tests indicated admissible deflections of 15.0–15.75 mm (*L*/40), supported by applied masses between 39.6 and 104.3 kg (≈388–1023 N) without rupture or permanent damage. Apparent flexural stresses ranged from 6.7 to 9.3 MPa, with apparent moduli between 0.7 and 1.9 GPa. All tiles achieved 100% approval in deformation, impact (2–8 J), and visual criteria. The results demonstrate that geometric effects dominate structural performance, validating miriti wood as an efficient and sustainable core for normatively compliant composite roofing systems.

## 1. Introduction

Composite materials have been increasingly adopted in civil engineering applications due to their favorable combination of low density, high mechanical performance, and design flexibility. In roofing systems, fiber-reinforced polymer composites offer advantages over traditional materials such as cementitious or metallic tiles, including reduced self-weight, improved durability, and enhanced resistance to environmental degradation. However, achieving structural efficiency while maintaining sustainability and normative compliance remains a key challenge, particularly when alternative core materials are introduced [1,2,3,4,5].

Sandwich composite structures represent an effective strategy to address this challenge. By combining stiff, load-bearing faces with lightweight cores, sandwich systems exploit geometric effects—especially the increase in second moment of area—to achieve high stiffness without a proportional increase in mass. In such configurations, structural performance is governed primarily by geometry and load transfer mechanisms rather than by the intrinsic strength of the core material. This concept has motivated the exploration of low-density and sustainable core materials as substitutes for conventional synthetic foams or honeycomb structures [6,7,8].

Conventional sandwich core materials widely adopted in structural applications include PVC foam, PET foam, end-grain balsa wood, and aluminum or polymeric honeycombs. These materials typically exhibit densities ranging from approximately 60 to 200 kg/m^3^ for polymeric foams, 120 to 160 kg/m^3^ for end-grain balsa, and even lower equivalent densities for honeycomb structures, depending on cell geometry. Their widespread use is primarily associated with controlled microstructures, predictable shear properties, and well-established mechanical databases. However, these engineered cores generally involve higher processing energy, industrial manufacturing steps, and, in some cases, limited regional availability. In contrast, miriti wood exhibits an exceptionally low density of approximately 90 kg/m^3^, positioning it within the lower bound of structural foam densities, while offering renewability, biodegradability, and regional sourcing advantages. The present study therefore does not aim to replace high-performance engineered cores in aerospace or marine applications, but to evaluate whether an ultra-light lignocellulosic core can provide sufficient geometry-driven stiffness for construction-oriented roofing systems under normative serviceability conditions.

Lignocellulosic materials have attracted growing interest as sustainable alternatives in composite systems due to their renewability, low density, and reduced environmental impact. Nevertheless, their application in structural components is often limited by concerns related to material heterogeneity, moisture absorption, and variability in mechanical properties. These concerns are particularly relevant in construction applications, where reliability, serviceability, and compliance with technical standards are critical. As a result, many studies focus on average mechanical properties, while variability, efficiency per unit mass, and normative conformity are seldom addressed in an integrated manner [9,10]. This gap highlights the need for studies that move beyond material characterization and address structural performance from a component-scale and standards-driven perspective.

Miriti wood, a lignocellulosic material abundantly available in the Amazon region, exhibits an extremely low specific gravity, making it a promising candidate for lightweight sandwich cores. Despite its favorable density characteristics, the structural potential of miriti wood in composite systems remains underexplored, especially when evaluated from a design-oriented and normative perspective. In particular, there is a lack of studies that simultaneously assess geometric efficiency, statistical variability, and compliance with construction standards, rather than relying solely on conventional strength-based metrics [11,12,13]. Such an approach requires shifting the focus from intrinsic material strength to structural configuration and performance validation under realistic service conditions.

In this context, the present research is guided by the following scientific questions:

(i) Can an ultra-low-density lignocellulosic core, such as miriti wood, provide sufficient structural performance when employed in a sandwich configuration governed primarily by geometric stiffness rather than intrinsic material strength?
(ii) To what extent does geometric amplification of the second moment of area compensate for the inherent variability and low density of natural cores under normative serviceability conditions?
(iii) Is the structural efficiency of such a system governed predominantly by material properties or by geometric configuration and laminate integrity?

Addressing these questions is essential for advancing the understanding of sustainable sandwich composites beyond material-level characterization, enabling component-scale evaluation under standardized construction requirements.

It is important to clarify the specific advancement of the present study in comparison with conventional low-density sandwich cores, such as polymeric foams and honeycomb structures. Although these materials also exhibit low densities, their application in construction-oriented sandwich systems is typically supported by material-level characterization or laboratory-scale panels, with limited emphasis on component-scale normative compliance. In contrast, the present work evaluates full-scale roofing tiles under standardized serviceability, impact, and visual criteria, demonstrating that an ultra-light lignocellulosic core can be successfully employed in normatively compliant structural elements.

Furthermore, unlike engineered foams and honeycombs that rely on controlled and uniform microstructures, miriti wood is a naturally heterogeneous material. The proposed sandwich architecture intentionally exploits geometry-driven stiffness—through increased section thickness and second moment of area—to decouple structural performance from intrinsic material variability. This design-oriented approach allows reliable deformation resistance and impact performance to be achieved without increasing material consumption or relying on highly processed synthetic cores.

From a sustainability perspective, the use of miriti wood also represents an advancement by combining low density with renewability, low embodied energy, and regional availability, while still meeting the mandatory requirements of ABNT NBR 16753 [14]. This alignment between sustainability objectives and normative compliance forms the central motivation of the present investigation. Therefore, the contribution of this research lies not in introducing a new low-density core per se, but in demonstrating that geometry-driven sandwich design enables sustainable, variability-tolerant, and normatively robust roofing systems suitable for real construction applications.

In addition, statistical treatment in composite research is frequently restricted to inferential analyses of mean values, which may be inadequate for prototype-scale structural elements and heterogeneous natural materials. Exploratory statistical approaches, mass-normalized performance metrics, trend analyses, and conformity-based assessments offer a more appropriate framework for interpreting the behavior of sandwich composites intended for real-world applications. When combined with normative testing, such approaches allow performance to be evaluated in terms of reliability and robustness, rather than isolated material optimization.

Within this context, the present study investigates sandwich-type composite roofing tiles composed of a miriti wood core and fiberglass-reinforced polymer faces. The tiles are manufactured by hand lay-up and evaluated according to ABNT NBR 16753 [14], encompassing deformation resistance, flexural behavior, water absorption, impact performance, and visual integrity. Beyond conventional characterization, the study adopts an integrated statistical framework that includes advanced descriptive statistics, exploratory correlations, mass-normalized performance indicators, trend analysis, and normative statistical assessment based on conformity.

The main objective of this work is to experimentally evaluate whether a miriti-based sandwich configuration can achieve structurally efficient and normatively robust performance, considering the intrinsic variability of lignocellulosic materials. It is important to emphasize that the present study does not presuppose the structural adequacy of miriti wood as a core material. Instead, the investigation is conducted under a performance-based and standards-driven framework, in which the feasibility of the proposed sandwich configuration is objectively verified through experimental testing and normative conformity assessment. The results are therefore interpreted in light of measured performance indicators, rather than predetermined assumptions.

## 2. Materials and Methods

### 2.1. Materials

This research proposes the development of a trapezoidal-profile roofing tile manufactured from a composite material composed of glass fibers, polymeric resin, and miriti wood. The selection of glass fiber as the primary reinforcement is justified by its well-established mechanical properties, including high tensile strength and elastic modulus, combined with relatively low cost, ease of processing, and widespread industrial use. Moreover, when compared with conventional construction materials that are heavier and exhibit reduced service life, glass fiber-reinforced composites offer superior structural efficiency and durability.

In parallel, the incorporation of miriti wood introduces a sustainable component to the composite system. Miriti is a renewable natural resource abundantly available in the Amazon region and exhibits significant potential for diverse engineering applications. Its use aligns with current global and industrial trends toward sustainability, which emphasize the valorization of regional materials, reduction in environmental impacts, and development of eco-efficient construction solutions.

The proposed material system consists of a sandwich composite structure formed by combining miriti wood with glass fiber-reinforced polymer laminates, specifically designed for the production of trapezoidal-profile roofing tiles. The raw materials used for the production of the roofing tiles were:

- Miriti wood, obtained in the municipality of Abaetetuba, Pará, Brazil, specifically from the islands of Sirituba and Maracapucu;
- Crystal polyester resin CENTERPOL–400;
- MEKP (methyl ethyl ketone peroxide), used as a curing initiator at a concentration of 1 wt%;
- Glass fiber fabric with an areal weight of 160 g/m^2^;
- Glass fiber mat with an areal weight of 300 g/m^2^.

**Figure 1:**
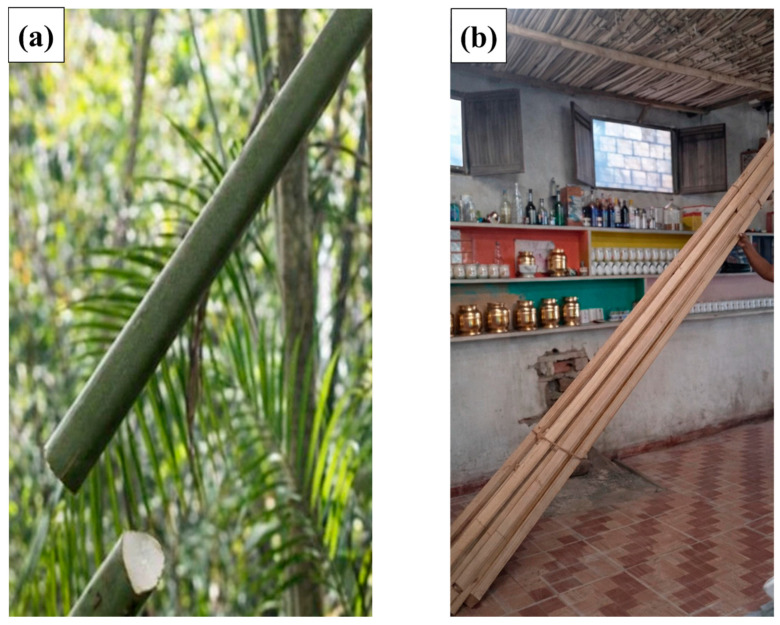
(**a**) Green petiole and (**b**) dry petiole.

The miriti wood employed in this study was extracted from the petiole of palm trees, as illustrated in Figure 1.

The glass fibers and polyester resin employed in this study are commercially available materials with well-established chemical compositions widely reported in the literature. Since no chemical modification, surface treatment, or interfacial functionalization was applied to these constituents, molecular-level characterization techniques such as FTIR spectroscopy were not performed. The scope of this work focuses on the structural, geometric, and normative performance of sandwich composite roofing elements at the component scale rather than on chemical or molecular-level material development.

### 2.2. Methods

#### 2.2.1. Preparation Process of Miriti Wood Panels

To ensure proper understanding and reproducibility of the experimental procedure, the preparation of miriti wood panels was carried out in a sequential manner. The process began with the extraction of the raw material from the petiole of the miriti palm tree. Initially, the outer layer, locally known as tala, was manually removed, and the material was subsequently subjected to natural sun drying for a period of 15 days in order to reduce moisture content and promote dimensional stabilization.

After this initial drying stage, the miriti pieces were transported to a local artisan workshop, where they underwent further processing. The material was cut into right prisms with a rectangular cross-section, presenting an average length of approximately 1500 mm and a base width of 35 mm. White polyvinyl acetate (PVA) adhesive was applied to the contact surfaces, and the assembled elements were placed in a mechanical press to ensure proper adhesion and consolidation of the panels.

Following the pressing stage, the bonded panels were again exposed to sunlight for approximately 4 h to accelerate the adhesive drying process. The total drying time required for complete curing was, on average, 12 h. After full drying, the panels were cut using a tensioned steel wire mounted on a worktable and supported by small wooden spacers, locally referred to as bitolas. This cutting procedure allowed the panels to be shaped into board-like elements with widths of 14 cm, 5.5 cm, 3.5 cm, and 1.5 cm, enabling proper adaptation to the trapezoidal geometry of the mold.

**Figure 2:**
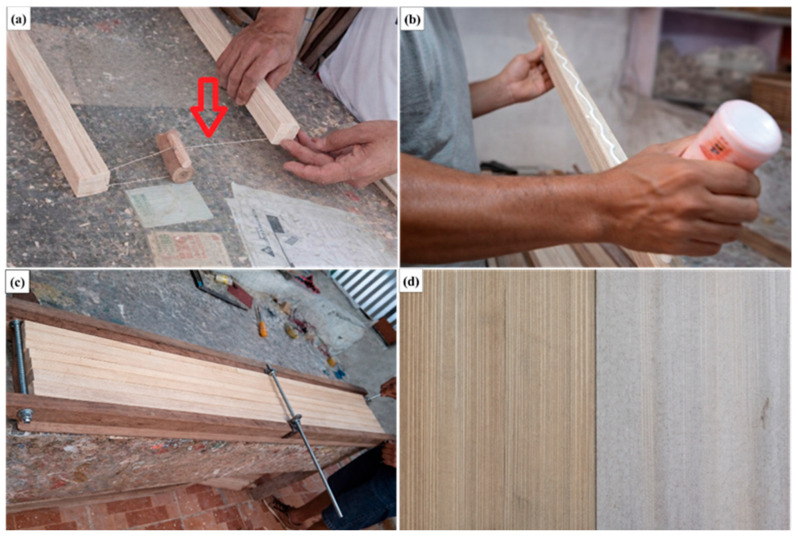
(**a**) Cutting the petioles into a right prism shape with a rectangular base, (**b**) gluing the miriti pieces together, (**c**) pressing to prepare the miriti wood, and (**d**) miriti wood.

The preparation of the raw material and the cutting process using the steel wire are illustrated in Figure 2a. The bonding procedure, in which adhesive was applied to only one bonding surface to avoid excessive accumulation at the joint and prolonged drying time, is shown in Figure 2b. For panel fabrication, four miriti elements with a square cross-section of 35 mm × 35 mm were assembled. To ensure adequate consolidation during bonding, a custom-made press composed of lateral wooden containment supports, threaded steel rods acting as tie bars, and steel clamps was employed, as illustrated in Figure 2c.

The miriti wood used in this study is presented in Figure 2d, where the darker-toned material corresponds to “terra firme” miriti, characterized by a denser and more fibrous structure, while the lighter-colored material corresponds to “várzea” miriti, which exhibits a more porous and spongy morphology. It is worth noting that, for the fabrication of the first roofing tile prototype, miriti panels with reduced dimensions of 500 mm × 500 mm and an average thickness of 8 mm were employed, as they were suitable for the initial molding and process evaluation stages.

#### 2.2.2. Manufacturing Process of the Roofing Tiles

**Figure 3:**
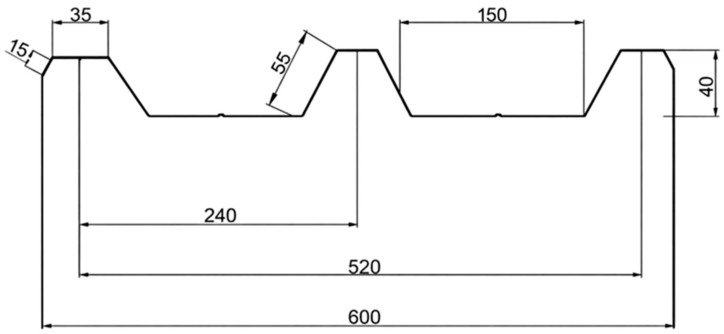
Profile of the manufactured tile (measurements in mm).

The trapezoidal-profile sandwich roofing tiles composed of miriti wood, glass fiber mat, and glass fiber fabric were manufactured at the Mechanical Manufacturing Laboratory of the Federal Institute of Pará (IFPA), Belém campus. The manufacturing procedure was carried out in accordance with the requirements of ABNT NBR 16753 [14]. With respect to the fabrication technique, the hand lay-up process was adopted due to its simplicity, versatility, and suitability for composite laminates with complex geometries. Initially, the geometric dimensions of the roofing tile profile were defined in order to comply with the dimensional criteria established by the standard, as well as the requirements for subsequent mechanical and physical characterizations. The profile dimensions selected for the fabrication of the roofing tiles are illustrated in Figure 3, which presents the trapezoidal geometry adopted for the composite sandwich structure.

Subsequently, the laminate architecture adopted for the manufacturing of the roofing tiles was defined, considering two distinct configurations. These architectures were intentionally selected to clarify the role of reinforcement arrangement on the structural and normative performance of the proposed miriti-based sandwich system. The objective was not to optimize laminate stacking sequences per se, but to assess whether the structural response of the roofing tiles is governed primarily by geometry-driven sandwich effects rather than by the specific surface reinforcement configuration.

The first configuration, referred to as Type I architecture, consists of alternating layers of glass fiber fabric and glass fiber mat as face reinforcements, with a central miriti wood layer acting as the core of the sandwich structure. This configuration results in higher resin uptake and greater mass per unit area and is expected to provide enhanced damage tolerance and improved stress redistribution.

**Figure 4:**
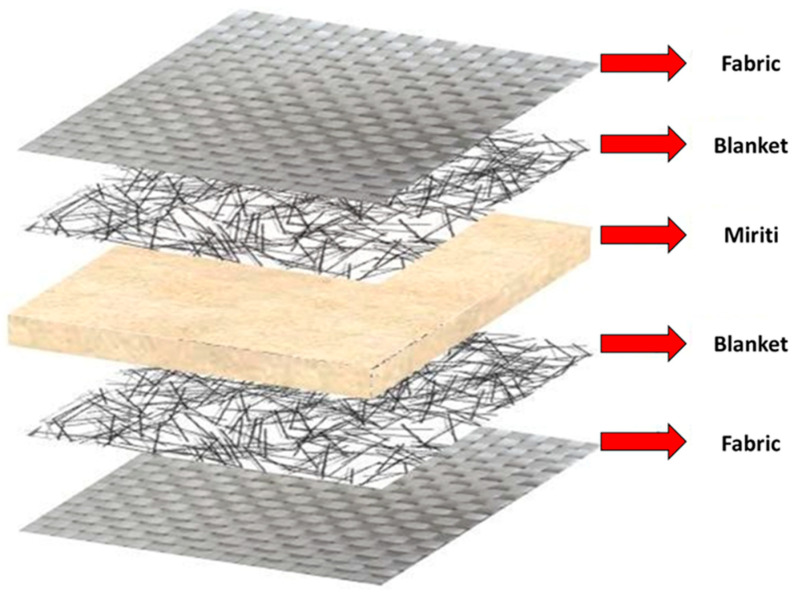
Type 1 architecture defined for the production of trapezoidal roof tiles.

The second configuration, referred to as Type II architecture, is composed exclusively of glass fiber fabric layers, also separated by a central miriti wood core. This architecture was designed to reduce resin consumption and overall weight while preserving structural integrity. By comparing these two laminate configurations under identical geometric constraints and standardized normative testing conditions, the study evaluates the sensitivity of deformation resistance, impact behavior, and stiffness-to-weight efficiency to the reinforcement architecture. Figure 4 illustrates the Type 1 architecture adopted for the production of the roofing tiles.

**Figure 5:**
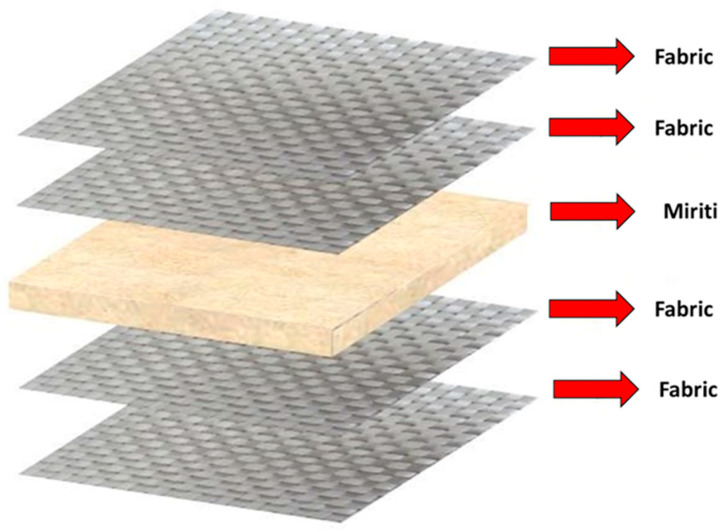
Type 2 architecture defined for the production of trapezoidal roof tiles.

Considering the high amount of resin absorbed by the glass fiber mat layers, new roofing tiles were manufactured using reinforcement composed exclusively of glass fiber fabric, with the objective of reducing the final mass of the product. This configuration prioritized a lighter sandwich structure while maintaining adequate structural integrity. Figure 5 illustrates the Type 2 architecture adopted for this purpose.

A commercially available trapezoidal-profile metal roofing tile, manufactured by Brasilit (São Paulo, Brazil), was used as the mold for the fabrication process. The mold presented dimensions of 1.80 m in length and 109 cm in width. The gel coat applied during the manufacturing of the composite roofing tiles was of the orthophthalic type, as it was formulated using orthophthalic polyester resin (CENTERPOL–400, Center Química, São Paulo, Brazil). The composition of the gel coat is presented in Table 1, where the masses of talc, titanium dioxide, and aerosil were calculated as percentages of the total mass of the polyester resin employed. The additives used in the formulation included industrial talc (Imerys Talc, Rio de Janeiro, Brazil), titanium dioxide (TiO_2_, Tronox Pigments, Botlek, The Netherlands), and fumed silica (Aerosil^®^, Evonik Industries, Essen, Germany). Methyl ethyl ketone peroxide (MEK-P) was used as the curing agent at a proportion of 1 wt% relative to the resin mass (Butanox M-50, Nouryon, Amsterdam, The Netherlands). All materials were commercially obtained from local distributors in Belém, Pará, Brazil.

**Figure 6:**
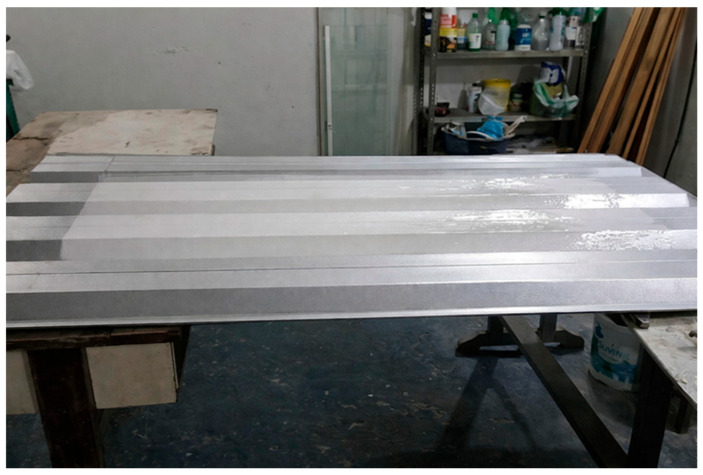
Gel coat application.

The manufacturing process began with the treatment of the mold using two layers of carnauba-based release wax to facilitate demolding. Subsequently, the gel coat was prepared and applied to the mold surface. Figure 6 illustrates the application of the gel coat on the trapezoidal roofing tile mold.

**Figure 7:**
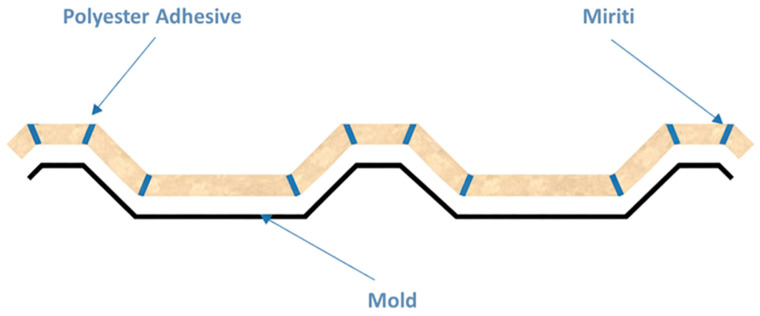
Shaping of miriti wood on the surface of the mold.

After approximately 1 h and 20 min following the application of the gel coat, the first two layers of glass fiber reinforcement were positioned on the mold surface and subsequently laminated. This step was followed by the placement of the miriti wood core, which was cut into custom-sized pieces corresponding to each section of the mold. The miriti elements were interconnected using an adhesive composed of the polyester matrix itself, as illustrated in Figure 7.

**Figure 8:**
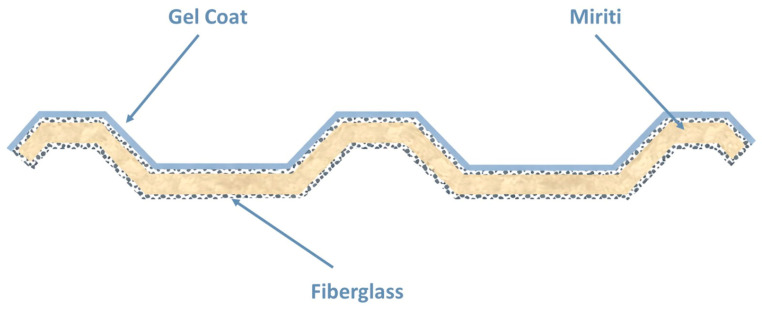
Schematic drawing of the cross-section of the manufactured tiles.

Finally, the last two layers of glass fiber reinforcement were applied, fully encapsulating the miriti wood core. This configuration was adopted to provide adequate structural stiffness to the roofing tile and to minimize water absorption by the miriti wood, which may exceed 200% within 24 h, according to Santos [15]. As a result, the manufactured roofing tiles exhibited a cross-sectional configuration consistent with the schematic representation shown in Figure 8.

For clarity and consistency throughout this manuscript, the term “Type” is used exclusively to designate the laminate architecture (Type I and Type II), while the term “Tile” refers to the individual roofing tile prototypes manufactured and tested (Tiles 1 to 5). Thus, multiple tiles may share the same architectural type but differ in dimensions, thickness, or constituent mass. Table 2 summarizes the composition of each of the five manufactured roofing tiles, detailing the mass of each constituent component. In addition, Table 2 also presents the mass of gel coat applied to the surface of the roofing tiles.

**Figure 9:**
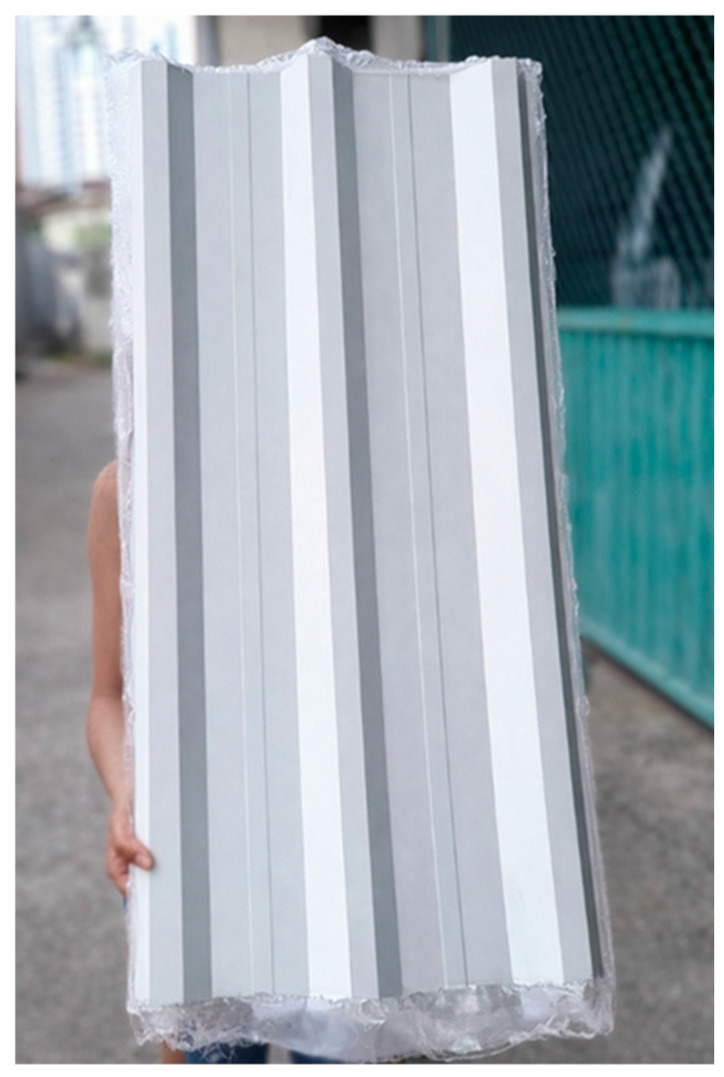
Manufactured trapezoidal miriti/fiberglass roof tile.

It can be observed that there is a variation in the mass of the miriti wood among the manufactured roofing tiles. This difference is attributed to the distinct types of miriti used in their fabrication, namely “Várzea” miriti and “terra firme” miriti, which present different densities and morphological characteristics. Figure 9 illustrates one of the miriti wood and glass fiber composite roofing tiles produced in this study.

#### 2.2.3. Classification of the Manufactured Roofing Tiles According to ABNT NBR 16753

According to ABNT NBR 16753 [14], fiber-reinforced polymer roofing tiles are classified based on a combination of geometric descriptors and mandatory performance requirements. The standard provides typical reference values for dimensions, thickness, and mass per unit area, which are intended to describe conventional monolithic laminate products commonly available on the market, while conformity and acceptance are ultimately established through compliance with mandatory mechanical, physical, and visual performance tests.

With respect to laminate type, roofing tiles may be classified as opaque, translucent, or opaque/translucent self-extinguishing, depending on their optical characteristics and fire-related behavior. The composite roofing tiles developed in this study fall into the opaque category. In terms of resistance to environmental exposure, ABNT NBR 16753 [14] defines Grade 1 tiles as those incorporating at least two protective layers with anti-UV additives and a gel coat, whereas Grade 2 tiles rely on anti-UV additives combined with a thermofused polyester film. All manufactured tiles incorporated a gel coat formulated with protective additives and therefore satisfy the requirements for Grade 1 classification.

Regarding dimensional characteristics, the standard establishes recommended maximum dimensions of up to 3200 mm (±6 mm) in width and up to 12,000 mm in length. All manufactured roofing tiles comply with these dimensional limits. The individual geometric dimensions of each tile, including width and length, are explicitly reported in Table 3.

ABNT NBR 16753 [14] also indicates typical values of mass per unit area ranging from 1.35 kg/m^2^ to 4.72 kg/m^2^, with an allowable variation of ±10%, depending on laminate configuration. These values are presented as reference ranges rather than strict acceptance limits. The manufactured sandwich roofing tiles fall entirely within this normative reference range, with individual values between 2.26 and 4.50 kg/m^2^, as explicitly reported in Table 3. Type I tiles exhibited higher mass per unit area (4.08–4.50 kg/m^2^), associated with thicker laminate build-up and the incorporation of glass fiber mat layers, whereas Type II tiles presented lower mass per unit area (2.26–2.37 kg/m^2^), reflecting a lighter sandwich configuration based on fabric-only laminates.

Concerning thickness, ABNT NBR 16753 [14] suggests typical laminate thickness values between 0.80 mm and 4.00 mm. These values correspond to monolithic fiberglass-reinforced polymer laminates and should be interpreted as informative references, not as mandatory geometric limits. In the present study, the manufactured roofing tiles exhibit total thickness values ranging from approximately 9.7 to 14.6 mm, as reported in Table 3. This increase in total thickness results from the intentional adoption of a sandwich composite configuration, in which thin fiberglass-reinforced polymer face laminates—whose individual thicknesses remain within the typical range indicated by the standard—are separated by a lightweight miriti wood core acting primarily as a geometric spacer.

It is important to emphasize that, in sandwich structures, the total section thickness does not represent the thickness of a single load-bearing laminate. Instead, the increased thickness is a design feature aimed at enhancing flexural stiffness through an increase in the second moment of area, rather than a deviation from accepted laminate manufacturing practice.

Ultimately, ABNT NBR 16753 [14] adopts a performance-based approach, in which acceptance is governed by compliance with mandatory serviceability and safety criteria, including deformation resistance, impact resistance, and visual integrity. Despite differing from the typical geometric characteristics of monolithic laminates, all manufactured sandwich roofing tiles fully satisfied these mandatory performance requirements, as demonstrated by the experimental results presented in subsequent sections.

Table 3 summarizes the classification and geometric characteristics of the manufactured roofing tiles according to ABNT NBR 16753 [14]. The results confirm that the proposed miriti-based sandwich roofing tiles comply with the mandatory performance criteria of the standard, while employing an alternative structural concept that extends the conventional application domain of fiberglass-reinforced polymer roofing elements.

#### 2.2.4. Characterization of the Roofing Tiles

The characterization of the trapezoidal-profile composite roofing tiles was carried out based on the requirements established by ABNT NBR 16753 [14], which was adopted as the reference standard for evaluating the performance of this type of product. This standard defines criteria related to the glass fiber content, mechanical properties, water absorption, visual aspects, as well as resistance to deformation and impact. In the present study, the experimental characterization was focused on the tests considered most representative of the service conditions and the objectives of the research. Accordingly, the glass fiber content, flexural properties, water absorption, visual aspects, resistance to deformation, and impact resistance were evaluated. The tensile test, although prescribed by the standard, was not performed, since composite roofing tiles exhibit a geometry and loading mode predominantly governed by flexural actions in typical applications, making flexural behavior the most relevant mechanical parameter for assessing their structural performance. Thus, the adopted approach ensures consistency between the normative requirements, the objectives of the study, and the practical application of composite roofing tiles, allowing for a technically sound evaluation of their mechanical and functional behavior. Table 4 summarizes the relevant geometric dimensions adopted in each experimental test, clarifying the distinction between the overall tile thickness, the local coupon thickness used in flexural testing, and the specimen dimensions employed in the water absorption test.

##### Visual Aspects

With respect to visual aspects, the laminates shall not exhibit stains, cracks, or voids, nor present surface fiber exposure or inclusions of foreign materials. In addition, the laminates must be free from surface wrinkles or undulations that may alter their nominal dimensions or compromise their geometric integrity. These requirements are essential to ensure not only the aesthetic quality of the product, but also its structural reliability and durability, since surface defects may act as stress concentrators and initiation sites for damage.

The evaluation of the manufactured roofing tiles was carried out by means of visual inspection. After the fabrication process, the roofing tiles underwent trimming and repair procedures using polyester resin, aiming to properly encapsulate the laminate core and to cover any localized glass fiber exposure. This post-processing step ensured the conformity of the roofing tiles with the visual requirements established by the applicable standard, contributing to the uniformity of the surface finish and the overall quality of the composite laminates.

##### Fiber Content

The ABNT NBR 16753 standard [14] recommends that the fiber content in composite laminates used for trapezoidal-profile roofing tiles shall be at least 25%. In this study, the fiber content was determined based on the mass fraction of glass fibers incorporated into the roofing tiles. The fiber content was calculated as the ratio between the mass of glass fibers applied during the manufacturing process and the total mass of the fabricated laminate, excluding the mass of the gel coat layer. This procedure allows for an accurate assessment of the effective reinforcement fraction within the structural laminate, ensuring consistency with the normative requirements and enabling a reliable evaluation of the material composition.

##### Determination of the Density of Miriti Wood

The density of miriti wood was determined following the procedure described by Santos [15], based on methods adapted from the ABNT NBR 7190 standard [16], which addresses the design and characterization of timber structures. The adopted methodology consisted of determining the density from the ratio between the oven-dry mass of the specimens and their corresponding geometric volume.

For this purpose, ten cubic miriti wood specimens were prepared, with nominal dimensions of 2 cm × 2 cm × 2 cm. The specimen dimensions were measured using a caliper, and their initial mass was determined using a precision analytical balance. Subsequently, the specimens were placed in an oven at a temperature of 103 ± 2 °C until mass stabilization was achieved, characterizing the oven-dry condition. After drying, the specimens were weighed again using the same precision balance.

Thereafter, the specimens were immersed in a beaker containing distilled water to determine the saturated mass, in accordance with the procedures established by ABNT NBR 7190 [16]. This approach enabled a reliable characterization of the density of miriti wood, ensuring the accuracy and reproducibility of the obtained results.

##### Water Absorption Test

The water absorption test was carried out at the IFPA–Abaetetuba Campus, following the general immersion and mass gain procedures established by ABNT NBR 14810 [17], which is commonly adopted as a reference for evaluating moisture uptake behavior in lignocellulosic-based materials and panels. In the present study, this standard was used as a methodological reference for immersion time, mass measurement, and calculation of water uptake, with adaptations introduced to reproduce the service condition of the developed roofing tiles.

A total of twenty specimens were prepared from Tile No. 1, manufactured with a miriti wood core and fiberglass-reinforced polyester faces. The specimens were extracted using a circular saw and presented nominal dimensions of 25 mm × 25 mm × 20 mm, which were verified using a caliper to ensure geometric accuracy prior to testing.

**Figure 10:**
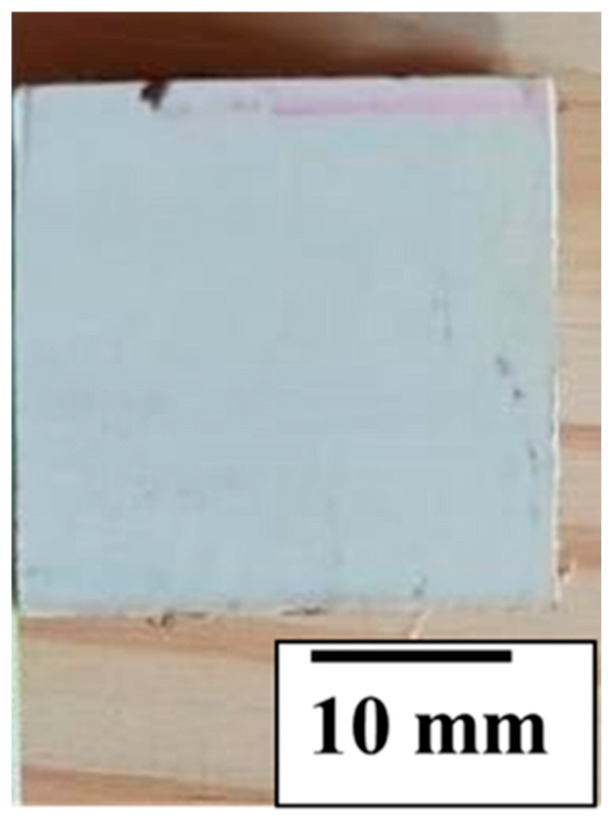
Measurement of test specimens for water absorption testing.

Due to the inherently high hygroscopicity of miriti wood and, more importantly, to ensure that the test realistically represented the actual service condition of the roofing tiles, the cut lateral surfaces exposing the miriti core were sealed with a polyester resin layer, ensuring complete encapsulation of the core. This procedure was intentionally adopted to prevent direct water ingress through the exposed edges and to reproduce the protective condition present in the manufactured roofing element, in which the miriti core is fully enclosed by polymeric faces and sealing resin. Figure 10 illustrates a representative specimen prepared for the water absorption test after cutting and sealing.

It is important to emphasize that, because of this sealing procedure, the present test does not quantify the intrinsic water absorption of raw miriti wood as a standalone lignocellulosic material. Instead, it quantifies the water uptake of the sealed sandwich-specimen system, under a service-representative configuration in which the core is protected by the composite faces and edge sealing. Therefore, the measured values should be interpreted as system-level water uptake rather than as material-level absorption of unprotected miriti wood.

After preparation and sealing, the specimens were weighed using a precision analytical balance to determine the initial dry mass (*M*_0_). Subsequently, the specimens were fully immersed in a container containing 1 L of distilled water, maintained at a controlled temperature of 20 °C. The immersion periods adopted were 2 h and 24 h, in accordance with the reference standard.

After the 2-h immersion period, the specimens were removed from the container and gently dried with absorbent paper to remove excess surface water, following a consistent and repeatable procedure. The specimens were then weighed to determine the saturated mass after 2 h of immersion. Subsequently, the specimens were re-immersed for an additional period, completing 24 h of total immersion, and weighed again following the same surface-drying procedure. The percentage water uptake was calculated using Equation (1).

(1) $$A=\frac{M_{1}-M_{0}}{M_{0}}\times 100$$

where

*A* = Water absorption (%);

*M*_1_ = Weight of the test specimen after the immersion period, in grams;

*M*_0_ = Weight of the test specimen before the immersion period, in grams.

##### Flexural Test (ASTM D790)–Apparent Flexural Response of Sandwich Specimens

The three-point flexural test was performed to evaluate the flexural response of the composite roofing tile specimens under controlled laboratory conditions. The test procedure followed the guidelines of ASTM D790 [18], which was adopted in this study as a practical and comparative flexural test, suitable for assessing the global flexural behavior of prototype-scale coupons extracted from the roofing tiles.

**Figure 11:**
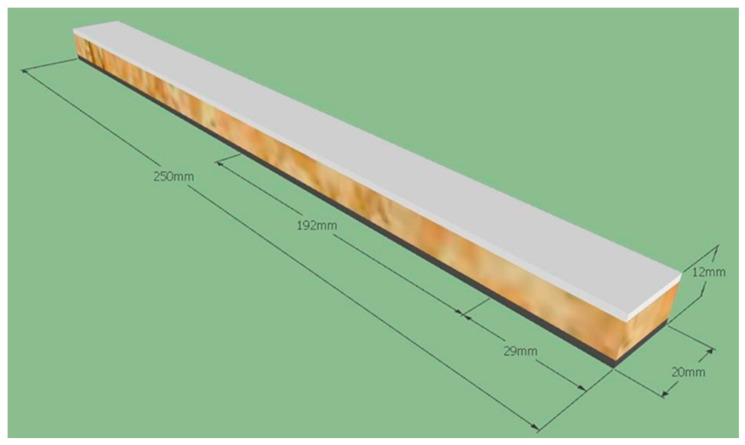
Dimensions of test specimens for flexural test.

A total of 20 specimens were obtained from Tile 1, and 5 specimens were extracted from each of Tiles 3, 4, and 5, resulting in a dataset representative of the different laminate architectures evaluated. All specimens presented a rectangular geometry, with nominal dimensions of 12 mm in thickness, 20 mm in width, and 250 mm in length (Figure 11). The support span was set to 192 mm, in accordance with ASTM D790 [18] recommendations, ensuring an adequate span-to-thickness ratio to promote flexural-dominated behavior.

The tests were conducted using an Arotec WDW-100E universal testing machine at a constant crosshead displacement rate of 5 mm/min. The equipment belongs to the Materials Characterization Laboratory of IFPA, Belém Campus (Belém, Brazil).

**Figure 12:**
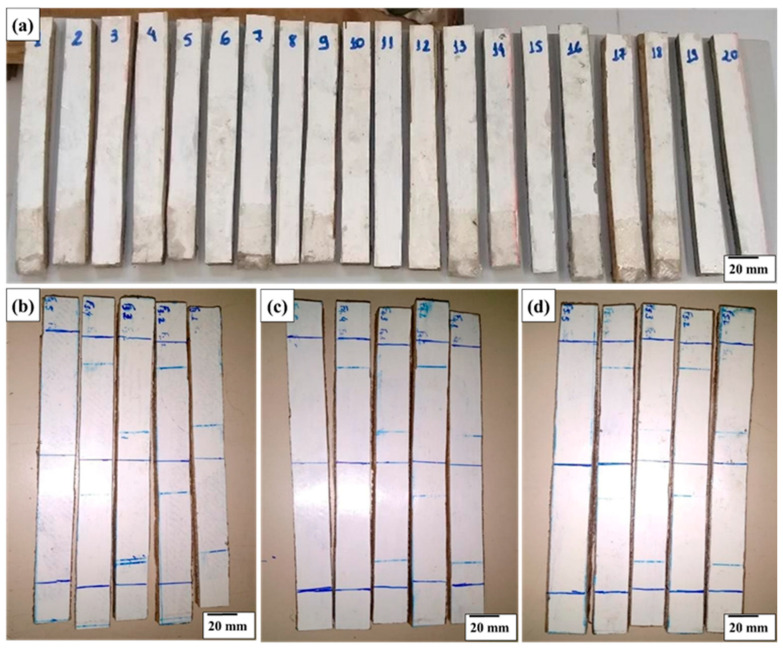
(**a**) Test specimens for flexural test (Tile 1), (**b**) Test specimens for flexural test (Tile 3), (**c**) Test specimens for flexural test (Tile 4) and (**d**) Test specimens for flexural test (Tile 5).

Figure 12 illustrates the test specimens extracted from tile numbers 1, 3, 4 and 5 for flexural strength testing.

**Figure 13:**
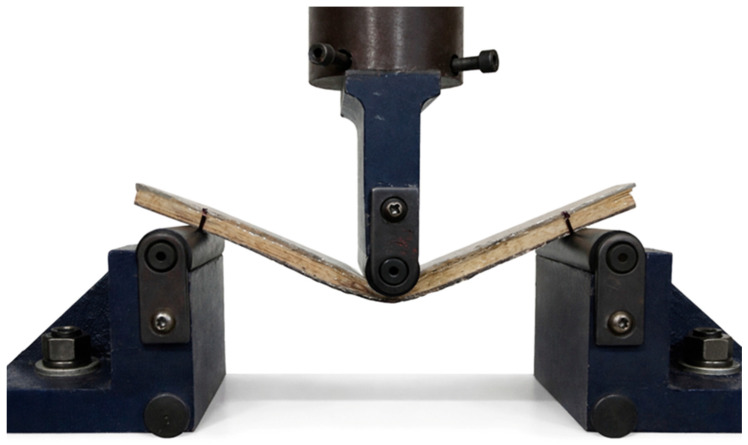
Test specimen being subjected to a flexural test.

The flexural tests were performed using an Arotec WDW-100E universal testing machine, operated at a crosshead speed of 5 mm/min. The equipment belongs to the Laboratory of Materials Characterization of the IFPA, Belém Campus (Belém, Brazil). Figure 13 shows the specimens during the execution of the flexural strength test.

It is important to emphasize that the tested coupons present a sandwich configuration, composed of a miriti wood core and fiberglass-reinforced polymer (GFRP) faces. In such structures, the stress and strain distributions under flexural differ fundamentally from those assumed in the classical homogeneous-beam formulation on which ASTM D790 [18] equations are based. In sandwich flexural, tensile and compressive stresses are carried predominantly by the faces, while the core contributes mainly through shear transfer and geometric separation of the faces.

Therefore, although the ASTM D790 [18] test configuration was employed, the conventional equations for stress, strain, and modulus derived for homogeneous rectangular beams do not represent the true material properties of the sandwich constituents. For this reason, all quantities calculated from this test are explicitly reported as apparent (nominal) flexural parameters, and they should be interpreted as global comparative indicators of flexural response under the adopted test configuration, rather than as intrinsic material properties.

For a rigorous identification of sandwich properties—such as facing stresses, core shear behavior, and flexural rigidity partition—dedicated standards including ASTM C393 [19] (flexural properties of sandwich constructions) and ASTM D7249 [20] (sandwich beam flexural stiffness and strength) are more appropriate and are recommended for future investigations.

The apparent (nominal) flexural stress was calculated using Equation (2), following the conventional ASTM D790 [18] formulation for rectangular beams and employed here solely as a comparative response metric:

(2) $$\sigma_{f}=\frac{3PL}{2bd^{2}}$$

where

*σ*_*f*_ is the apparent (nominal) flexural stress;

*P* is the applied load;

*L* is the span length between the supports;

*b* is the specimen width;

*d* is the specimen thickness.

The apparent flexural strain was calculated using Equation (3).

(3) $$\epsilon_{f}=\frac{6Dd}{L^{2}}$$

where

*ϵ*_*f*_ is the apparent flexural strain;

*D* is the displacement of the specimen during the test.

The apparent flexural modulus was determined from the slope of the initial linear portion of the apparent stress–strain curve using Equation (4).

(4) $$E_{f}=\left(\sigma_{f2}-\sigma_{f1}\right)/\left(\varepsilon_{f2}-\varepsilon_{f1}\right)$$

where

$E_{f}$ is the apparent flexural modulus;

$\sigma_{f2}$ and $\sigma_{f1}$ are the apparent flexural stresses measured at two points within the initial linear region of the curve;

$\varepsilon_{f2}$ and $\varepsilon_{f1}$ are the corresponding apparent flexural strain values.

The resulting apparent parameters were used exclusively for comparative analysis among different tile configurations, allowing qualitative assessment of stiffness, deformation capacity, and damage tolerance under flexural conditions, without implying direct material property equivalence with homogeneous composites. It should be noted that flexural strain is a dimensionless parameter, calculated as the ratio between displacement and span length. The notation mm/mm is mathematically consistent but represents a dimensionless quantity. For clarity, strain values are presented herein as dimensionless parameters. Therefore, ASTM D790 [18] was adopted as a comparative global response method consistent with prototype-scale evaluation, while sandwich-specific standards are recommended for future detailed mechanical characterization.

##### Deformation Resistance Test

The deformation resistance test was carried out in accordance with the procedure established in Annex A of ABNT NBR 16753 [14], which evaluates the flexural behavior of roofing tiles subjected to a uniformly distributed load under service-like conditions. The objective of this test is to assess the stiffness of the roofing element by determining the load required to reach a prescribed admissible deflection limit, without inducing rupture or permanent damage.

According to the standard, the admissible deflection limit is defined as *L*/40, where L corresponds to the effective clear span between the support lines, and not to the total length of the loading platform. Although the loading table employed in the test presents a total length of 1100 mm, the effective span considered for deflection control is governed by the distance between the supports, excluding the widths of the supports themselves and the load transfer regions imposed by the rigid loading platform.

In the experimental configuration adopted in this study, the effective clear span was approximately 600 mm, with slight variations between specimens due to differences in tile width and positioning. As a result, the admissible deflection limits calculated according to the *L*/40 criterion ranged from approximately 15.0 mm to 15.75 mm, values that are fully consistent with the requirements of ABNT NBR 16753 [14].

The test setup consisted of four wooden supports with a cross-section of 100 mm × 50 mm and a length of 700 mm, slightly greater than the width of the tested roofing tiles. To ensure uniform stress distribution and to minimize local stress concentrations, the upper surfaces of the supports were lined with a felt layer approximately 5 mm thick. A rigid wooden loading platform measuring 230 mm in width and 1100 mm in length was positioned centrally over the supports. The lower surface of the platform was also lined with felt of approximately 5 mm thickness to promote uniform load transfer along the effective span.

**Figure 14:**
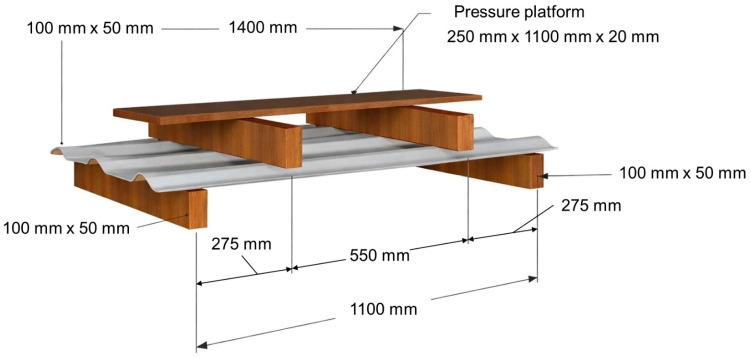
Apparatus for testing the resistance to deformation of roof tiles according to NBR 16753 [14].

A schematic representation of the test apparatus, including the support configuration, loading platform, and specimen positioning, is shown in Figure 14, highlighting the effective clear span used for deflection control in accordance with the standard.

For each test, the uniformly distributed load was applied incrementally by placing calibrated masses on the loading platform until the admissible deflection limit (*L*/40) was reached. The total applied mass at this condition, expressed in kilograms (kg), was recorded as the deformation resistance of the roofing tile. A total of five roofing tiles were tested, as prescribed by ABNT NBR 16753 [14].

**Figure 15:**
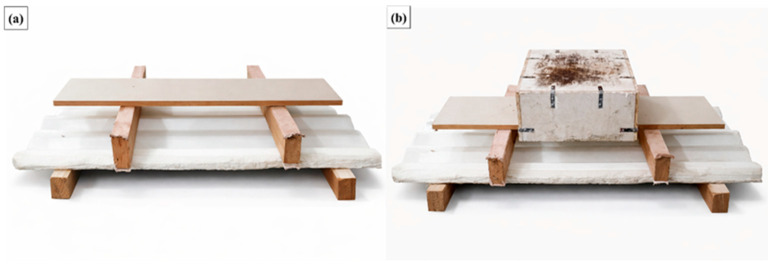
(**a**) Flexural test (equipment and tile specimen) and (**b**) tile specimen being tested.

Figure 15a illustrates a roofing tile properly positioned on the test apparatus prior to loading, while Figure 15b shows the application of the calibrated masses on the loading platform during the test. Throughout the loading process, no rupture, cracking, or permanent deformation was observed in any of the tested specimens, confirming that the tiles reached the serviceability deflection limit under stable structural conditions.

In accordance with ABNT NBR 16753 [14], the uniformly distributed load was applied by placing calibrated masses on the loading platform. Therefore, the experimental results are initially expressed in terms of applied mass (kg). For mechanical interpretation and discussion purposes, the corresponding equivalent force can be obtained by converting mass to force using the gravitational acceleration, according to F = mg, 9.81 m/s^2^.

##### Impact Test

The impact test was conducted using equipment developed in accordance with the guidelines established by ABNT NBR 16753 [14], at the Manufacturing Laboratory of the Federal Institute of Pará (IFPA), Belém Campus. This test aimed to evaluate the impact resistance of the roofing tiles, simulating dynamic loading conditions that may occur during service.

**Figure 16:**
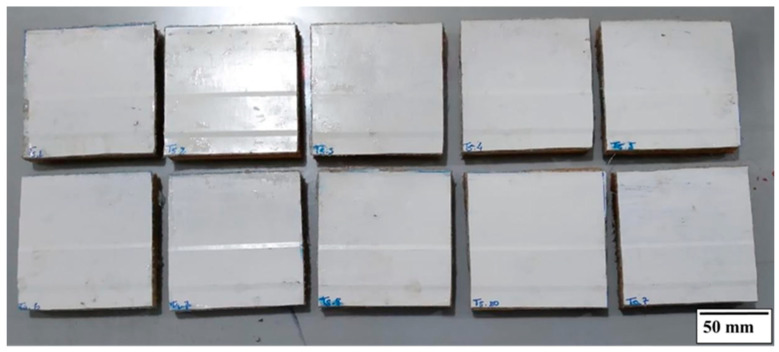
Test specimens for impact testing.

The specimens, as illustrated in Figure 16, were prepared with nominal dimensions of 100 mm × 100 mm, in compliance with the standard specifications. A total of 50 specimens were produced, consisting of 10 specimens from each type of roofing tiles evaluated, ensuring adequate statistical representativeness for the impact performance assessment.

Specimen cutting was performed using an electric saw (Makita 4100NH2, Makita Corporation, Anjo, Aichi, Japan), ensuring dimensional accuracy and appropriate edge finishing, thereby minimizing the introduction of defects that could adversely affect the impact test results.

The equipment used for the impact test consists of the following components, designed and assembled in accordance with the applicable standard requirements:

- Guide tube, with an effective travel length of 1 m, through which the impact bar—previously loaded with the corresponding weights that define the test mass—slides freely and delivers the impact to the striker positioned above the specimen;
- Impact bar and steel disc weights, used to assemble the total mass applied during the test. The individual disc weights have masses of 62.5 g, 125 g, 250 g, and 500 g, allowing different mass combinations and, consequently, different impact energy levels;
- Impact striker, manufactured from hardened steel, with a rounded tip and a diameter of (15.80 ± 0.10) mm, responsible for concentrating and transferring the impact energy to the specimen in a controlled and repeatable manner;
- Standard support plate for the specimen, measuring 110 mm × 110 mm × 35 mm, featuring a central hole with a diameter of (16.26 ± 0.025) mm. This plate is mounted on the lower base of the system and provides proper support for the specimen during the test;
- Guide tube and striker support, designed to hold the guide tube firmly in a vertical position, ensuring correct alignment and centering of the impact striker relative to the specimen.

**Figure 17:**
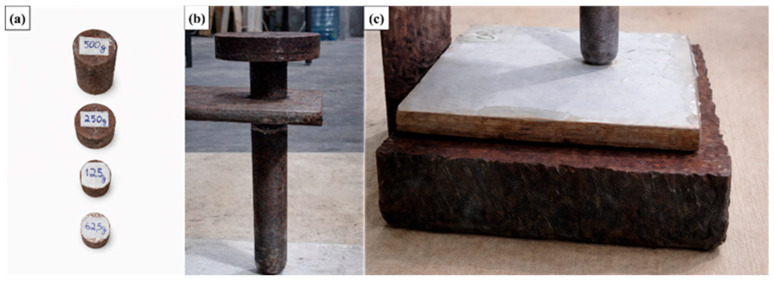
(**a**) Masses of weights on steel discs, (**b**) impact tip and (**c**) standard support plate for test specimens.

Figure 17 illustrates the equipment used in the impact test, as well as its configuration during test execution.

**Figure 18:**
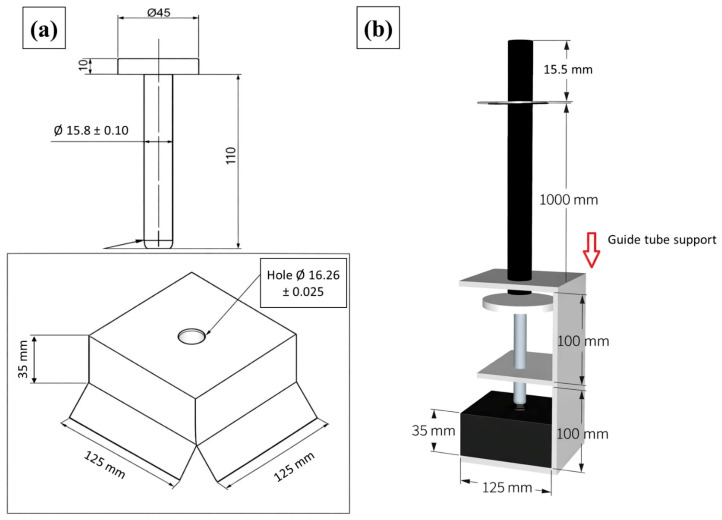
(**a**) Dimensional detailing of the percussion cap and support block, including geometric tolerances of the guide hole and (**b**) schematic view of the assembled unit, showing the guide tube, the tube support, the mass system and the main dimensions of the equipment used to perform the impact tests.

Figure 18 shows the schematic diagram of the impact test apparatus in accordance with the ABNT NBR 16753 standard [14].

**Figure 19:**
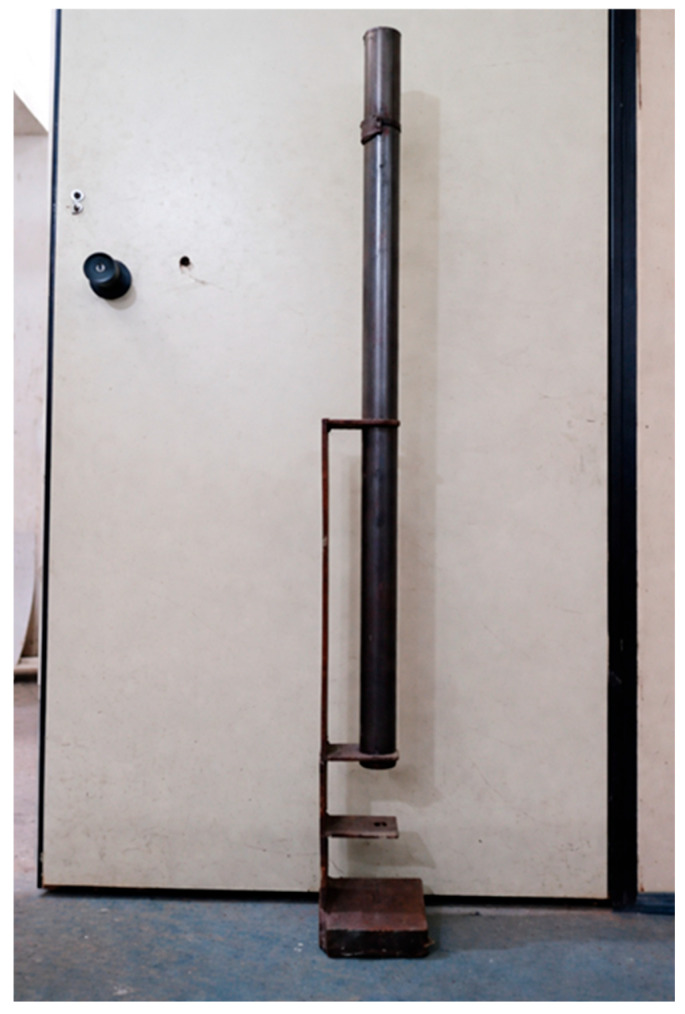
Equipment built for impact testing.

Figure 19 illustrates the equipment constructed in accordance with the standard parameters for the impact resistance test of the manufactured roofing tiles.

For the test procedure, it is first necessary to determine and identify the number of specimens for each sample group to be evaluated. Subsequently, the test apparatus is prepared by loading it with the predetermined mass corresponding to the test conditions to be applied. After the assembly and verification of the loading system, the impact energy is calculated according to Equation (5).

(5) E=m×g×h

where

*E* is the impact energy, expressed in joules (J);

*m* is the mass, expressed in kilograms (kg);

*g* is the acceleration due to gravity (m/s^2^);

*h* is the height, expressed in meters (m);

In the experimental setup adopted in this study, the drop height h was kept constant and defined by the effective travel length of the guide tube, equal to 1.0 m, in accordance with the recommendations of ABNT NBR 16753 [14]. Different impact energy levels (2.0, 3.7, 5.0, and 8.0 J) were therefore obtained by varying the total mass m of the impactor assembly through the combination of calibrated steel disc weights, while maintaining a constant drop height.

#### 2.2.5. Statistical Treatment of Experimental Data

To ensure a rigorous and coherent interpretation of the experimental results, a statistical treatment consistent with the structural, prototypical, and normative nature of the investigated composite roofing tiles was adopted. Given the limited number of specimens per configuration, the inherent heterogeneity of lignocellulosic materials, and the dominance of geometric effects in sandwich structures, the analysis was intentionally focused on descriptive and exploratory statistical methods, rather than classical inferential statistics. Due to the limited number of full-scale tile prototypes per architectural configuration and the absence of strictly controlled single-variable experimental grouping, inferential hypothesis testing between Type I and Type II architectures was not conducted. The statistical treatment was therefore intentionally restricted to descriptive, exploratory, and conformity-based approaches consistent with prototype-scale evaluation.

##### Descriptive Statistics

For all physical and mechanical parameters evaluated—including density, fiber content, water uptake, apparent flexural properties, deformation resistance, and impact response—the following descriptive statistical measures were calculated: arithmetic mean, standard deviation, coefficient of variation (*CV*) and minimum and maximum values.

The arithmetic mean ($\bar{x}$) was calculated as Equation (6).

(6) $$\bar{x}=\frac{1}{n}\sum_{i=1}^{n}x_{i}$$

where *x_i_* represents an individual experimental measurement and *n* is the number of tested specimens. The standard deviation (*s*) was calculated according to Equation (7).

(7) $$s=\sqrt{\frac{1}{n-1}\sum_{i=1}^{n}{\left(x_{i}-\bar{x}\right)}^{2}}$$

The coefficient of variation (*CV*), used as a normalized indicator of dispersion and repeatability, was calculated as Equation (8).

(8) $$CV\left(\%\right)=\frac{S}{\bar{x}}\times 100$$

The coefficient of variation was employed as a central metric to quantify the degree of variability associated with natural material heterogeneity, manual manufacturing processes, and geometric dispersion.

##### Exploratory Correlation Analysis

To identify the dominant parameters governing the structural performance of the sandwich roofing tiles, exploratory correlation analyses were conducted between key variables, including: Total tile thickness and admissible load at the serviceability limit (*L*/40); Mass per unit area and deformation resistance; Core density and deformation resistance.

The Pearson linear correlation coefficient (*r*) was calculated as Equation (9).

(9) $$r=\frac{\sum_{i=1}^{n}\left(x_{i}-\bar{x})(y_{i}-\bar{y}\right)}{\sqrt{\sum_{i=1}^{n}{\left(x_{i}-\bar{x}\right)}^{2}}\;\sqrt{\sum_{i=1}^{n}{\left(y_{i}-\bar{y}\right)}^{2}}}$$

where *x* and *y* represent the paired variables under analysis.

The correlation analysis was employed strictly in an exploratory and interpretative sense, without hypothesis testing or *p*-value estimation, given the limited sample size and the non-randomized nature of the specimens.

##### Mass-Normalized Performance Metrics

To allow fair comparisons between roofing tiles with different geometries and constituent masses, selected performance parameters were normalized by mass or mass per unit area. The mass-normalized deformation resistance (*P_m_*) was calculated as Equation (10).

(10) $$P_{m}=\frac{P}{m}$$

where *P* is the applied load required to reach the admissible deflection limit and m is the total mass of the roofing tile.

This normalization enabled the evaluation of stiffness-to-weight efficiency, which is particularly relevant for lightweight sandwich structures intended for roofing applications.

##### Normative Statistical Assessment

In addition to conventional descriptive statistics, a normative statistical approach based on conformity was adopted. For each standardized test prescribed by ABNT NBR 16753 [14], the approval rate was quantified as Equation (11).

(11) $$Approval\;rate\;\left(\%\right)=\frac{N_{approved}}{N_{tested}}\times 100$$

where *N_approved_* corresponds to the number of specimens meeting the normative requirements and *N_tested_* is the total number of tested specimens.

This conformity-based assessment provides a statistically meaningful measure of structural reliability and robustness at the component level, particularly suitable for construction-oriented composite products.

## 3. Results and Discussion

### 3.1. Visual Aspects

The visual inspection of the manufactured roofing tiles did not reveal the presence of surface cracks, stains, or voids, either on the surface or within the bulk of the material, indicating satisfactory quality of the manufacturing process. Any glass fiber protrusions resulting from the molding process were subsequently fully encapsulated by the polyester matrix during the finishing stage, ensuring surface integrity and visual uniformity of the final product.

Due to the incorporation of white additives into the gel coat formulation, the manufactured roofing tiles exhibited a white and opaque appearance on the upper surface. Visual evaluation conducted at a distance of 3 m, as specified by the applicable standard, indicated a highly uniform surface, free of perceptible discontinuities or defects, thus fully complying with the normative aesthetic requirements.

**Figure 20:**
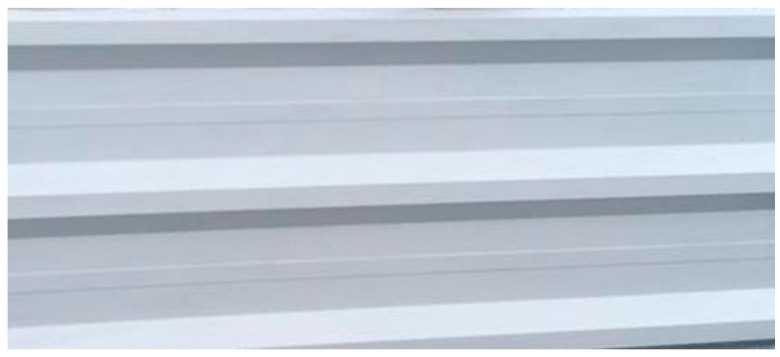
Upper surface of the trapezoidal miriti/fiberglass roof tile.

Figure 20 illustrates the surface of one of the evaluated roofing tiles, highlighting the quality of the surface finish and corroborating the observations obtained during the visual inspection.

### 3.2. Fiber Content

Table 5 shows the fiberglass content achieved in each manufactured tile.

The results presented in Table 5 indicate that the tiles manufactured with both architectures reached fiberglass contents close to the average value of approximately 30%, demonstrating the viability of the manufacturing process in incorporating the reinforcement in a globally consistent manner. However, a non-negligible dispersion is observed in the individual values, especially for the Type II architecture, which showed a more pronounced variation among the analyzed samples. This dispersion suggests the influence of variables inherent to the production process, such as local differences in compaction, resin distribution, and thickness control, aspects that are particularly sensitive in curved geometries such as tiles. From a statistical point of view, the reduced number of test specimens per architecture limits a conclusive comparison between the groups, so that punctual differences in fiber content should be interpreted with caution. Nevertheless, the results indicate that both architectures operate within a reinforcement content range compatible with structural applications in glass fiber reinforced polymer composites, although the observed variability highlights the importance of rigorous control of manufacturing parameters to ensure greater repeatability and reduce the influence of fiber content as an interfering variable in the evaluation of subsequent mechanical and physical properties.

### 3.3. Specific Gravity of Miriti Wood

Table 6 summarizes the results of the specific gravity determination carried out on miriti wood specimens following the procedure adapted from ABNT NBR 7190 [16].

Based on ten specimens, the material exhibited an average specific gravity of 0.091 g/cm^3^, confirming the extremely lightweight nature of miriti wood when compared to conventional structural timbers and other lignocellulosic materials commonly employed in engineering applications. In addition to the low mean value, the results reveal a non-negligible dispersion, with a standard deviation of 0.008 g/cm^3^ and a corresponding coefficient of variation (CV) of approximately 8.8%. The measured specific gravity values ranged from 0.078 to 0.105 g/cm^3^, evidencing the intrinsic heterogeneity of the material. This level of variability is consistent with what is typically reported for natural lignocellulosic materials and can be attributed to factors such as differences in anatomical structure, porosity distribution, local density variations along the palm petiole, residual moisture effects, and natural growth-related heterogeneities.

From an engineering standpoint, the extremely low specific gravity of miriti wood has direct and ambivalent implications for its application as a core material in sandwich composites. On one hand, it represents a significant structural advantage, as the reduction in density directly contributes to lowering the self-weight of the roofing tiles, facilitating handling, transportation, and installation. More importantly, when employed as a core in sandwich structures, the low-density miriti significantly increases the separation between the fiberglass-reinforced faces, thereby enhancing the second moment of area of the cross-section without a proportional increase in mass. This geometric effect is fundamental to the stiffness gains observed in the deformation resistance tests discussed in subsequent sections.

On the other hand, the low specific gravity of miriti wood is inherently associated with limited intrinsic strength of the solid material, reinforcing the fact that the core is not intended to act as a primary load-bearing component. Instead, its structural role is to provide geometric stability, shear transfer, and energy absorption, while the fiberglass-reinforced polymer faces carry the dominant tensile and compressive stresses under flexural. In this context, the observed dispersion in specific gravity values, although relevant from a materials characterization perspective, is expected to have a secondary influence on the global structural performance of the sandwich tiles when compared to parameters such as total thickness, face laminate integrity, and bonding quality.

The presence of measurable variability nevertheless highlights the importance of adequate material selection and quality control when using miriti wood in engineering applications. However, as demonstrated by the full compliance of the manufactured roofing tiles with the requirements of ABNT NBR 16753 [14], the sandwich architecture adopted in this study effectively mitigates the effects of material-level heterogeneity. By relying on geometry-driven stiffness and face-dominated load transfer mechanisms, the proposed composite system accommodates the natural variability of the miriti core while maintaining reliable structural and normative performance.

### 3.4. Water Uptake of Sealed Sandwich Specimens (Miriti-Core Composite)

**Figure 21:**
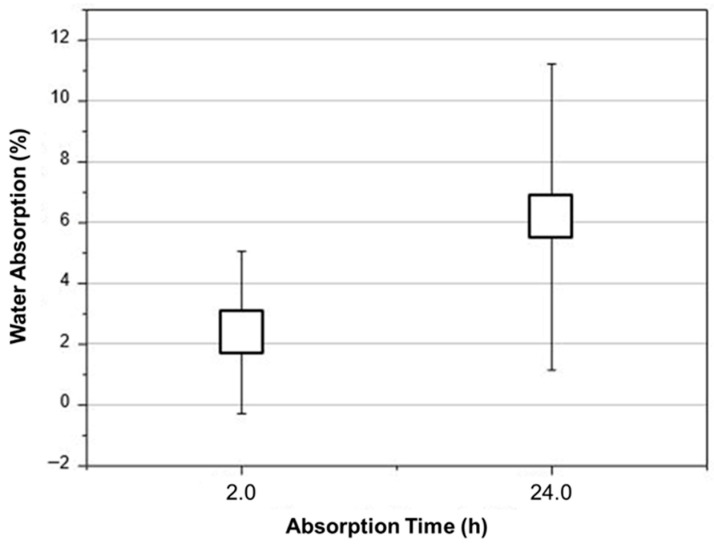
Graph showing the percentage absorption of water by miriti wood at 2 and 24 h.

Figure 21 presents the percentage mass gain measured in the immersion test performed on specimens extracted from Tile 1, whose cut lateral surfaces were sealed with polyester resin. Because the miriti core was encapsulated by the fiberglass-reinforced faces and the exposed edges were sealed, the reported values represent the water uptake of the sealed sandwich-specimen system, not the intrinsic water absorption of raw miriti wood.

This distinction is critical for interpretation: while unsealed miriti wood can exhibit very high-water absorption when directly exposed (e.g., values exceeding 200% after 24 h reported by Santos [15] for miriti without encapsulation), the present results quantify the effectiveness of the sandwich architecture and sealing strategy in limiting water ingress under a service-representative condition.

The graph of percentage water absorption of miriti wood (according to ABNT NBR 14810 [17]) indicates a time-dependent absorption behavior, with a clear increase between 2 h and 24 h, and also shows an important change in the variability of the results. At 2 h, absorption is concentrated in low values, with the “box” (interquartile range) situated approximately between ~2% and ~3%, and a median around ~2.5%. The “whiskers” extend to a slightly wider range (approx. from ~0% to ~5%), suggesting that, although most samples show low absorption in the short term, there are differences between specimens attributable to the natural heterogeneity of the material (local porosity, density variation, fiber/vessel orientation, microcracks and initial moisture). Even so, the overall picture at 2 h is one of moderate and relatively well-controlled absorption, with contained dispersion.

Within 24 h, there is a clear shift in the distribution towards higher values: the box is positioned approximately between ~5.5% and ~6.8%, with a median around ~6%, that is, more than double the typical value observed in 2 h. This increase is physically expected because, initially, water tends to penetrate rapidly by capillarity into more accessible pores and channels, while, over time, diffusion progresses to less accessible regions and voids are filled more widely, in addition to the gradual increase in water-cell wall interaction (cellulose/hemicellulose hydroxyls). The critical point, however, is that the dispersion in 24 h becomes much greater: the whiskers extend from approximately ~1% to ~11%, indicating that some samples absorbed little water even in 24 h, while others absorbed a significant amount. This increased variability in the long term is a sign that the phenomenon is becoming strongly controlled by microstructural characteristics and specific defects of each sample, such as: the presence of more open vessels, a higher fraction of lumens, preferential permeability paths, density variation, discontinuities (cracks, natural delaminations), as well as possible differences in surface finish (roughness) and in the drying/conditioning conditions before the test.

From a critical standpoint, this graph allows for two complementary interpretations. The first is positive: the central (median) values at 2 h and 24 h remain within a relatively low range (on the order of a few percent), suggesting that, on average, miriti does not exhibit extreme absorption under the test conditions—something relevant when considering applications where dimensional stability and susceptibility to moisture are concerns. The second interpretation is a technical warning: the upper tail at 24 h (samples reaching ~10–11%) indicates that there is a subset of specimens with high permeability/effective hygroscopicity, which, in engineering practice, means a risk of less predictable behavior. In lignocellulosic materials, predictability is as important as the average value, because failures due to moisture and degradation are usually governed by “worst-case scenarios” (more porous or more defective samples), especially in real service with wetting/drying cycles.

Another point that deserves methodological clarification (and which often explains dispersion) is that absorption results according to standards for lignocellulosic panels/composites are quite sensitive to preconditioning (initial moisture and reference dry mass), water temperature control, actual immersion time, and the procedure for removing excess surface water before weighing. If, for example, surface water removal is not standardized (same absorbent paper pressure, same time), this can artificially increase variability, especially at 24 h, when surfaces may retain more water.

Although the average water uptake values of approximately 2.5% after 2 h and 6.0% after 24 h are relatively low for a lignocellulosic-based system, their repeatability was further assessed through the coefficient of variation (CV). For the sealed sandwich specimens, the water absorption after 2 h exhibited a low to moderate dispersion, with CV values on the order of approximately 20–25%, indicating good short-term repeatability and controlled moisture ingress. After 24 h of immersion, the dispersion increased, with CV values reaching approximately 40–45%, reflecting the growing influence of microstructural heterogeneity, porosity distribution, and localized permeability paths within the miriti core.

This increase in variability with immersion time is characteristic of natural lignocellulosic materials and does not indicate a loss of structural reliability. Importantly, despite the higher CV observed at 24 h, the absolute absorption values remained moderate and did not compromise deformation resistance, impact performance, or normative compliance. These results confirm that the encapsulated sandwich configuration effectively limits moisture ingress while maintaining repeatable system-level behavior.

#### Sealed Sandwich Water Uptake Versus Mechanical Integrity: Overcoming a Common Misconception

Lignocellulosic materials are often assumed to be mechanically unreliable in humid environments due to their hygroscopic character. However, the present results indicate that such a generalization does not necessarily apply to properly designed and encapsulated sandwich composites. The water uptake results obtained for sealed sandwich specimens (ABNT NBR 14810 [17]) show a time-dependent increase, with median values remaining in a moderate range after 24 h. Although some dispersion is observed—expected for natural porous materials—this absorption behavior must be interpreted in light of the actual structural configuration adopted in the roofing tiles.

In the manufactured tiles, the miriti core is not exposed as a free surface; instead, it is encapsulated by fiberglass-reinforced polyester faces and sealing resin at cut surfaces, which significantly reduces direct water ingress pathways into the core during service. This detail is critical: the water absorption test confirms the intrinsic affinity of miriti for moisture, but the tile design deliberately mitigates this effect through polymeric encapsulation.

Most importantly, when the absorption behavior is analyzed alongside the mechanical and normative outcomes, the results contradict the common prejudice that “natural cores inevitably compromise structural performance.” Despite the hygroscopic nature of miriti, the tiles fully complied with ABNT NBR 16753 [14] serviceability criteria in the deformation resistance test and exhibited no structural failure in the impact test. This indicates that, for the evaluated conditions, moderate moisture uptake at the material level does not translate into loss of mechanical integrity at the component level, provided that the sandwich architecture ensures face-dominated load carrying and the core remains adequately protected.

From a structural mechanics viewpoint, this finding is coherent: in sandwich action, the faces govern tensile/compressive resistance and the core governs stability and shear transfer. Therefore, even if the core experiences some moisture-related mass change, the normative performance can remain stable as long as (i) face properties and bonding integrity are preserved and (ii) the core remains encapsulated. These results support the feasibility of miriti as a sustainable core for composite roofing applications and suggest that engineering design (encapsulation and sandwich integrity) is the decisive factor in overcoming moisture-related concerns typically associated with lignocellulosic materials.

Finally, although the present study demonstrates normative compliance and mechanical integrity under the adopted conditions, further investigations under conditioning protocols (wet–dry cycles and long-term exposure) are recommended to quantify durability and to establish performance envelopes for harsher environmental scenarios. In addition to cyclic conditioning, future investigations should include mechanical testing after environmental aging to quantify potential reductions in flexural stiffness, deformation resistance, and impact performance, particularly in relation to face/core interface integrity. Nevertheless, the present results represent short-term immersion behavior. Long-term durability evaluation under extended immersion periods and cyclic wet–dry conditioning is recommended for future investigations to assess potential interfacial degradation and long-term moisture effects.

### 3.5. Composite Flexural Test

The flexural test results presented in Table 7 are reported in terms of apparent (nominal) flexural stress, apparent flexural strain, and apparent flexural modulus, calculated using the conventional ASTM D790 [18] rectangular-beam formulation. Because the tested coupons have a sandwich architecture (miriti core + GFRP faces), these values should be interpreted as global comparative indicators of flexural response under the adopted test configuration, rather than as intrinsic material properties. In sandwich flexural, tensile and compressive stresses are primarily carried by the faces, whereas the core contributes mainly through shear transfer and geometric separation of the faces; therefore, direct comparison with homogeneous GFRP laminates or bulk materials must be made with caution.

The apparent flexural properties summarized in Table 7 indicate distinct differences in the mechanical behavior of the investigated roofing tile configurations. As expected for sandwich-type specimens tested using a conventional ASTM D790 [18] formulation, the reported values must be interpreted as global, comparative indicators of flexural behavior rather than intrinsic material properties of the constituent phases.

In terms of apparent flexural stress, the maximum values ranged from approximately 6.7 to 9.3 MPa. Type II tiles, particularly Tile 3, exhibited the highest apparent flexural stress, indicating a stiffer response and a greater resistance to initial bending loads. In contrast, Tile 1, belonging to the Type I architecture, reached a lower peak stress, suggesting a reduced initial stiffness and earlier onset of damage mechanisms under flexural loading.

The apparent flexural deformation values highlight a markedly different mechanical behavior. While Tiles 3, 4, and 5 exhibited limited deformation capacity, with apparent flexural deformation concentrated between approximately 1.1 and 1.5%, Tile 1 demonstrated a significantly higher deformation capacity, reaching values close to 3.0%. In contrast to the other configurations, Tile 1 exhibited a more gradual post-peak response, maintaining residual stress over a broader deformation range.

This contrasting behavior indicates distinct damage mechanisms governing the flexural response. The Type II tiles exhibited a more brittle or quasi-brittle response, characterized by higher peak stress followed by a relatively abrupt loss of load-carrying capacity, consistent with localized interlaminar shear or unstable delamination. Conversely, the Type I configuration showed a more progressive damage evolution, likely associated with matrix cracking, localized core crushing, and gradual interfacial debonding, which enabled continued load transfer after the peak stress.

The apparent flexural modulus values further support this interpretation. Type II tiles presented higher apparent moduli, ranging from approximately 1.2 to 1.9 GPa, reflecting a stiffer structural response dominated by the fiberglass fabric layers. Tile 1 exhibited a significantly lower apparent modulus (≈0.7 GPa), consistent with its lower initial stiffness and greater deformation tolerance. The relatively large dispersion observed in the modulus values, particularly for Tile 3, can be attributed to geometric variability, local thickness differences, impregnation quality, and the inherent limitations of applying homogeneous-beam formulations to sandwich specimens.

For interpretative purposes, the apparent flexural stress (6.7–9.3 MPa) and apparent flexural modulus (0.7–1.9 GPa) obtained in this study can be contextualized by comparison with sandwich flexural data reported in the literature using dedicated standards such as ASTM C393 [19] and ASTM D7249 [20]. Studies on polymer-based sandwich panels with lightweight cores typically report facing-dominated flexural stresses on the order of 10–40 MPa and equivalent flexural stiffness values ranging from approximately 1 to 10 GPa, depending on face material, core thickness, and span configuration.

Although direct quantitative comparison is not appropriate due to differences in test configuration, specimen geometry, and data reduction methods, the present apparent values fall within the lower bound of ranges reported for sandwich structures with ultra-light cores. This behavior is consistent with the use of an extremely low-density lignocellulosic core and thin GFRP faces, confirming that the measured apparent flexural response reflects geometry-controlled sandwich action rather than intrinsic material limitations. Therefore, the reported nominal values are physically coherent with sandwich mechanics and comparable in order of magnitude to results obtained using sandwich-specific flexural standards.

It is important to emphasize that the observed trends cannot be explained solely by differences in fiberglass content. Despite similar reinforcement fractions among the tiles, the flexural response varied substantially, indicating that laminate architecture, thickness distribution, and manufacturing-induced defects play a more dominant role than reinforcement content alone. This observation reinforces the concept that, for sandwich structures with ultralight cores, geometric effects and load-transfer mechanisms govern flexural behavior more strongly than isolated material properties.

From a structural design perspective, the flexural results suggest a trade-off between stiffness and deformation capacity. Type II configurations provide higher stiffness and peak resistance, whereas the Type I configuration offers enhanced deformation tolerance and damage accommodation. Although Tile 1 exhibits lower apparent flexural strength, its ability to sustain significant deformation without catastrophic failure may be advantageous in roofing applications, where energy dissipation, serviceability, and damage tolerance under distributed loads are critical performance criteria.

**Figure 22:**
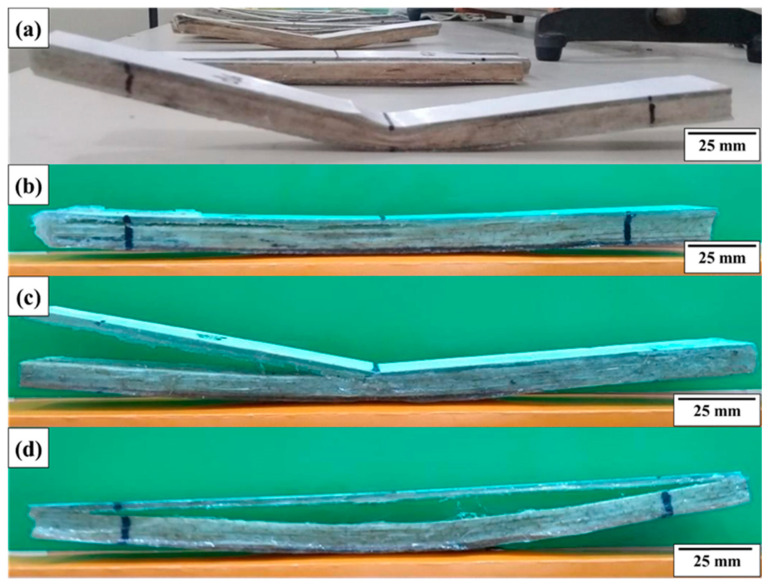
(**a**) core crushing, (**b**–**d**) detachment of the upper lamina.

Figure 22 illustrates the tile 1 test specimens used for the flexural test, after the load was applied, showing the detachment of some of the upper layers.

**Figure 23:**
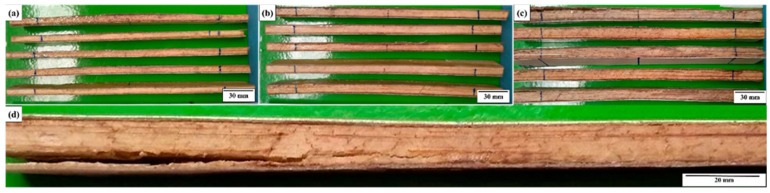
(**a**) Test specimens extracted from tile 3 after the tests were carried out; (**b**) Test specimens corresponding to tile 4; (**c**) Test specimens obtained from tile 5; and (**d**) Enlarged detail showing a specific case of debonding of the lower layer of the composite laminate, observed in a small number of samples.

Figure 23 shows the visual aspect of the specimens extracted from tiles 3, 4, and 5 after the mechanical tests, allowing a qualitative assessment of the damage mechanisms induced by the applied load. In general, it is observed that the specimens maintained their structural integrity, since the identified damage was minimal. Visual inspection revealed only a slight and practically imperceptible crushing deformation of the core in some samples, without evidence of severe failures. As highlighted in Figure 23d, the detachment of the lower layer of the laminate occurred in a localized manner and in a limited number of specimens, not characterizing a predominant failure mechanism. These results indicate that the composite system showed good structural performance, preserving interlaminar adhesion and core stability even after the application of mechanical loading.

**Figure 24:**
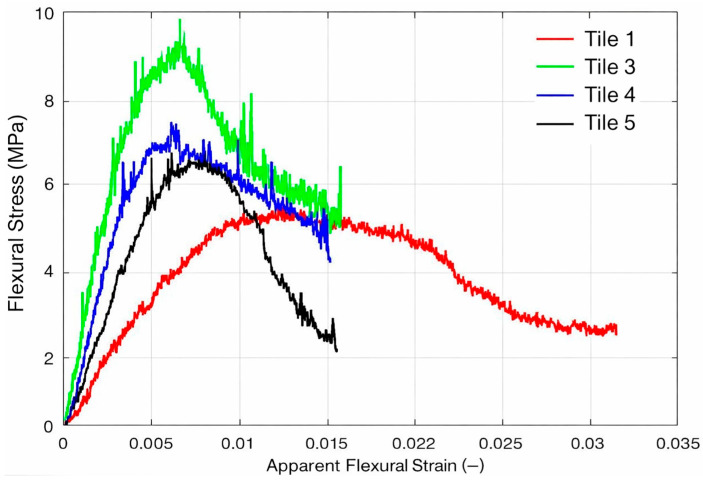
Flexural stress–strain curves of the test specimens.

Figure 24 illustrates the stress versus strain behavior of typical specimens from each series taken from tiles 1, 3, 4, and 5, tested in flexural.

The flexural stress–strain diagram clearly shows that the tiles not only reach different strength levels, but also fail through different mechanisms, with quite contrasting post-peak responses (softening). In general terms, Tile 3 (green curve) exhibits the highest peak stress, reaching approximately 9–10 MPa around ε ≈ 0.005–0.007 mm/mm, followed by a gradual and irregular drop in stress, typical of progressive damage (microcracking of the matrix, nucleation and propagation of delaminations, and load redistribution between regions of the laminate). Tile 4 (blue) reaches a lower peak, around 7–7.5 MPa at similar deformations (ε ≈ 0.005–0.007), and also exhibits a relatively smooth post-peak, suggesting that, despite failing at lower stress than Tile 3, it still retains some resistance capacity after the onset of damage, which is consistent with a more distributed degradation process. Tile 5 (black), on the other hand, exhibits a critical characteristic: it grows to approximately 6.5–6.8 MPa and, after reaching its maximum, suffers an abrupt drop around ε ≈ 0.010–0.012, with rapid loss of resistance capacity, behavior consistent with a more “catastrophic” failure event, frequently associated with unstable delamination, interlaminar shear, localized rupture due to defects (voids, resin-rich region) or even crushing/instability on the compressed side with sudden collapse of the effective section.

Tile 1 (red) is the most unique of the set: its initial section has a lower slope (lower apparent stiffness), grows more gradually, and reaches a relatively low peak, close to 5.0–5.3 MPa, but at a higher deformation (ε ≈ 0.012–0.015). After the peak, Tile 1 exhibits a long tail until approximately ε ≈ 0.03–0.032, maintaining residual stresses on the order of 2.5–3 MPa over a wide deformation range. This shape is typical of a material with a higher deformation capacity before global rupture, in which damage accumulates gradually and the structure still “loads” through mechanisms such as interlaminar friction, partial fiber spotting, and stress redistribution in still intact regions. In other words, although Tile 1 is the worst in terms of maximum stress, it may be the most relevant when the design criteria involve damage tolerance and energy absorption capacity, because the energy dissipated in flexural is proportional to the area under the curve: a curve with a lower peak, but with a prolonged post-peak, can absorb total energy comparable to or even greater than a curve with a high peak and a rapid drop.

From a critical point of view, the set of curves also reveals that the observed variability cannot be attributed solely to “differences in architecture,” as there are strong indications that manufacturing quality and internal integrity are dominating the response. The oscillations and “serrations” (especially in Tile 3) suggest successive damage events (microcracks/delaminations) and/or measurement noise; however, when these serrations are very intense, they can also indicate local discontinuities (porosity, thickness variations, matrix-rich regions) that lead to micro-collapses and load reorganization throughout the test. Furthermore, the difference in initial stiffness between the tiles (Tiles 3 and 4 being stiffer; Tile 1 being less stiff) points to variations in parameters that directly affect the flexural modulus, such as the actual thickness of the specimen (in flexural, small variations in thickness generate large variations in the calculated stress), span/thickness ratio, alignment at the supports, and, mainly, voids and impregnation. This helps explain why tiles with relatively similar fiber content can exhibit such distinct responses: in real structures, small differences in fiber fraction can be overcome by larger differences in defects, fiber-matrix adhesion, fabric waviness, and pre-existing delamination.

#### Failure Mode Analysis in Sandwich Flexure

The flexural response observed in the tested specimens can be further interpreted within the framework of classical sandwich failure mechanisms. In sandwich structures subjected to three-point bending, several failure modes may occur, including tensile rupture of the bottom face, compressive failure or wrinkling of the top face, core shear failure, core crushing under the loading nose, and face/core debonding.

In the present study, visual inspection of the failed specimens (Figure 22 and Figure 23) indicates that the dominant damage mechanisms were localized core crushing beneath the loading nose and progressive interlaminar debonding of the face laminates. No evidence of global core shear failure across the span was observed, which suggests that the miriti core, despite its low density, provided sufficient shear transfer capacity under the tested span-to-thickness ratio.

In Tile 1 (Type I architecture), the extended post-peak behavior and sustained residual stress indicate progressive damage mechanisms, likely associated with matrix cracking, localized core indentation, and gradual face/core debonding. The absence of abrupt load drops suggests that catastrophic tensile face rupture or unstable face wrinkling did not govern the failure process. Instead, damage evolved progressively, which is consistent with ductile energy-dissipative behavior in sandwich systems with compliant cores.

In contrast, Tile 5 exhibited a sharper post-peak load drop, which may be associated with localized debonding between the lower face and the core or unstable interlaminar shear failure. However, even in these cases, complete tensile rupture of the fiberglass face was not observed, and structural integrity was largely maintained.

No clear evidence of classical face wrinkling instability was detected in the compressed upper faces. This is likely attributed to the relatively small span and moderate compressive stresses induced under the adopted ASTM D790 [18] configuration. The failure patterns observed therefore indicate that global flexural collapse was governed primarily by local core crushing and interface degradation rather than by face instability or pure core shear failure.

These findings are consistent with sandwich mechanics theory, in which ultralight cores primarily act as shear-transfer media and geometric spacers, while the faces carry the dominant tensile and compressive stresses. The absence of catastrophic shear failure or face rupture further supports the suitability of miriti wood as a compliant yet structurally stable core for geometry-driven sandwich applications.

### 3.6. Deformation Resistance Test

Table 8 summarizes the results of the deformation resistance test of the composite tiles, expressed by the total load required for each test specimen to reach the maximum allowable deflection, previously defined as a function of the width of each tile (*L*/40 criterion), according to ABNT NBR 16753 [14]. It should be emphasized that the admissible deflection values reported in Table 8 are based on the effective clear span of the test configuration, as defined by the standard, rather than the total length of the loading platform.

A significant variation in the supported load is observed between the tiles analyzed, with values ranging from approximately 39.6 kg (Tile 3) to 104.3 kg (Tile 1), highlighting marked differences in structural rigidity between the manufactured prototypes.

Tile 1 demonstrated the best performance, supporting the highest load before reaching its limit deflection, which can be directly associated with its more robust structural architecture, composed of combined layers of fabric and fiberglass mat associated with a miriti core. This configuration favors a better distribution of stresses and greater efficiency in load transfer between the laminate faces and the core, a typical characteristic of well-consolidated sandwich-type structures. Furthermore, the relatively high average thickness of this tile contributes to increasing the moment of inertia of the cross-section, a determining factor for flexural stiffness.

In contrast, Tile 3 exhibited the lowest load supported to achieve the same allowable deflection order, despite meeting the normative criteria. This behavior can be attributed to its lower average thickness, as well as to the lamination architecture composed exclusively of fiberglass fabric, without the contribution of the mat, which tends to reduce the capacity to accommodate interlaminar stresses and the overall stiffness of the system. This result reinforces that, although all tiles were approved according to the normative criteria, there are significant differences in structural performance that should be considered when evaluating practical applications.

Tiles 4 and 5 exhibited intermediate behavior, supporting loads of approximately 49.2 kg and 64.3 kg, respectively. These values reflect a balance between thickness, reinforcement architecture, and geometry, indicating that small variations in these parameters can result in substantial changes in the final stiffness of the tile. It is important to emphasize that, although the allowable deflection varies slightly between the test specimens due to differences in width, this variation is relatively small and does not, by itself, explain the observed dispersion in load values, reinforcing the predominance of structural and constructive effects.

From a statistical and methodological point of view, it should be considered that the results represent the overall behavior of tiles manufactured by a manual process, in which the variability inherent in the conformation of the miriti core, the impregnation of the fibers, and the final thickness of the laminate can significantly influence the mechanical performance. Even so, the fact that all tiles reached the limit deflection without showing rupture or permanent deformation demonstrates the efficiency of the structural concept adopted and the viability of using miriti wood as a core in composite tiles.

To further reinforce the stiffness-to-weight efficiency of the proposed sandwich configuration, the deformation resistance results were normalized by the mass per unit area of each roofing tile. When expressed in terms of supported load per unit mass, the tiles exhibited load-to-mass ratios ranging from approximately 15.5 to 27.2 kg/kg. In particular, lighter Type II tiles, despite sustaining lower absolute loads, achieved equal or superior mass-normalized performance compared to heavier Type I configurations.

This normalization highlights that structural efficiency is not governed solely by the maximum admissible load, but by the interaction between geometry, total thickness, and self-weight. The results demonstrate that the miriti-based sandwich architecture achieves high deformation resistance with reduced mass per unit area, confirming that the observed performance gains arise from geometry-driven stiffness rather than increased material consumption.

Table 9 presents a comparison between the flexural test results obtained in this work for the composite tile reinforced with miriti wood and fiberglass and those reported by Oliveira [21] for cementitious tiles reinforced with natural fibers, with and without the addition of brick residue.

It is observed that the miriti tile developed in this study withstood a significantly higher maximum load, reaching 104.29 kg without rupture, while the comparative cementitious tiles showed structural rupture under loads of 60 kg (without residue) and 85 kg (with 50% brick residue). In addition to the higher load capacity, the miriti tile showed a maximum deflection of only 15 mm, a value substantially lower than the deflections recorded in the cementitious tiles (37 mm and 48 mm, respectively), demonstrating a more rigid and stable structural behavior under flexural stress.

This superior performance can be attributed mainly to the sandwich architecture of the composite, in which the miriti wood core efficiently absorbs and redistributes stresses, while the outer fiberglass layers significantly contribute to increasing the flexural stiffness and overall strength of the system. Unlike cementitious tiles, which exhibit predominantly brittle behavior and abrupt rupture, the miriti/fiberglass composite demonstrated a more ductile behavior, with greater capacity for controlled deformation before failure, a desirable characteristic in roofing elements subjected to distributed loads and accidental actions.

However, it is important to emphasize that the comparison between the studies should be analyzed with caution, since they involve materials of different natures, different manufacturing processes, and non-equivalent failure mechanisms. Furthermore, Table 9 presents the best results obtained in this work, not directly reflecting the statistical dispersion observed among the tested samples, which indicates the need for complementary analyses with a larger number of specimens to evaluate the variability of mechanical performance. Even so, considering these limitations, the results consistently indicate that the composite tile with a miriti core presents a relevant structural advantage, associating greater load capacity, less deflection, and absence of rupture, when compared to traditional solutions reported in the literature.

For contextual comparison, conventional monolithic glass fiber reinforced polymer (GFRP) roofing tiles compliant with ABNT NBR 16753 [14] typically present mass per unit area values in the range of approximately 3.5–4.7 kg/m^2^, as indicated by the normative reference values and by commercially available products. In contrast, the miriti-based sandwich tiles developed in this study exhibited mass per unit area between 2.26 and 4.50 kg/m^2^, with Type II configurations concentrated near the lower bound of this range (≈2.3 kg/m^2^).

In terms of deformation resistance, monolithic GFRP roofing tiles generally satisfy the *L*/40 serviceability criterion with admissible loads primarily governed by laminate thickness, but without benefiting from the geometric stiffening associated with sandwich architectures. The present sandwich tiles reached the admissible deflection limit under applied masses ranging from 39.6 to 104.3 kg, demonstrating that comparable or superior deformation resistance can be achieved at significantly reduced mass per unit area. This comparison highlights the efficiency gains provided by the sandwich configuration, in which increased section thickness and moment of inertia are obtained without a proportional increase in self-weight.

For contextual benchmarking, the deformation resistance and stiffness-to-weight performance of the developed miriti-based sandwich tiles can be qualitatively compared with literature data on sandwich panels employing conventional cores such as PVC foam, PET foam, and end-grain balsa. Structural PVC foams with densities between 80 and 120 kg/m^3^ typically provide core shear moduli in the range of 20–50 MPa and are widely used in lightweight sandwich panels for marine and civil applications. End-grain balsa cores, with densities around 120–160 kg/m^3^, exhibit higher compressive strength and shear stiffness, enabling superior absolute load capacity but at increased mass. Honeycomb cores offer excellent stiffness-to-weight ratios but require more complex manufacturing and precise bonding conditions.

Although direct quantitative comparison is not strictly possible due to differences in geometry, span configuration, and test methodology, the present results demonstrate that miriti-based sandwich tiles achieve deformation resistance levels comparable in order of magnitude to lightweight foam-core systems used in non-structural building envelopes, while maintaining mass per unit area values within or below the typical range of monolithic GFRP roofing products. Importantly, the observed structural performance was achieved without the use of synthetic foams or industrially engineered cores, relying instead on geometry-driven stiffness amplification and face-dominated load transfer.

Therefore, miriti wood should not be interpreted as a direct substitute for high-performance aerospace or marine sandwich cores, but rather as a viable alternative for construction-scale sandwich elements where serviceability, mass efficiency, sustainability, and normative compliance are the governing design criteria.

#### 3.6.1. Quantitative Comparison with Conventional GFRP Roofing Tiles

In addition to cementitious roofing systems, it is important to contextualize the performance of the developed miriti-based sandwich tiles relative to conventional monolithic glass fiber reinforced polymer (GFRP) roofing tiles commercially available in the market and compliant with ABNT NBR 16753 [14].

Typical monolithic GFRP roofing tiles exhibit mass per unit area values ranging from approximately 3.5 to 4.7 kg/m^2^, depending on laminate thickness and reinforcement content. Their deformation resistance under the *L*/40 serviceability criterion is primarily governed by laminate thickness and resin/fiber ratio, without geometric stiffening from a separated core.

In the present study, the miriti-based sandwich tiles exhibited mass per unit area values between 2.26 and 4.50 kg/m^2^. Notably, Type II configurations achieved values close to 2.3 kg/m^2^, significantly below the lower bound commonly reported for monolithic GFRP tiles. Despite this reduced mass, the sandwich tiles sustained admissible loads between 39.6 and 104.3 kg without rupture, demonstrating comparable or superior deformation resistance relative to monolithic systems operating at higher mass.

When normalized by mass, the load-to-mass ratio of the developed sandwich tiles reached up to 27.2 kg/kg, indicating enhanced stiffness-to-weight efficiency attributable to geometric amplification of the second moment of area. This comparison highlights that the proposed sandwich architecture achieves structural performance levels consistent with conventional composite roofing systems while offering potential reductions in self-weight.

#### 3.6.2. Cross-Correlations Between Core Density, Thickness and Structural Performance

While the previous sections presented the physical and mechanical results of the composite roofing tiles individually, a more comprehensive understanding of the structural behavior emerges when these parameters are analyzed in a correlated manner. In sandwich-type structures, performance is not governed by isolated material properties, but by the interaction between core density, total thickness, and laminate architecture. Therefore, cross-correlations among these variables are essential to properly interpret the efficiency of the developed system.

The extremely low specific gravity of miriti wood (≈0.09 g/cm^3^), as determined in Section 3.3, would conventionally suggest limited structural applicability if the material were used as a load-bearing element. However, in the proposed sandwich configuration, miriti does not act as a primary stress-carrying component. Instead, its function is to increase the separation between the fiberglass-reinforced faces, thereby significantly enhancing the second moment of area of the cross-section without a proportional increase in mass. This geometric effect is a fundamental characteristic of sandwich structures and plays a dominant role in determining flexural stiffness.

This mechanism is clearly reflected in the deformation resistance results discussed in Section 3.6. Despite the low density of the core, the composite tiles were able to sustain relatively high, uniformly distributed loads before reaching the allowable deflection limit (*L*/40), as prescribed by ABNT NBR 16753 [14]. When analyzed from a stiffness-to-weight perspective, the results demonstrate that the lightweight miriti core effectively compensates for its limited intrinsic strength through geometric stiffening, resulting in elevated structural efficiency per unit mass.

A direct correlation is also observed between the total thickness of the tiles and the maximum load required to reach the admissible deflection. Tiles with greater total thickness consistently supported higher loads, even when differences in fiberglass content and laminate architecture were relatively small. This behavior is fully consistent with classical flexural theory, in which flexural stiffness scales strongly with thickness due to its cubic influence on the second moment of area. In this context, variations in thickness—primarily associated with differences in miriti core dimensions and laminate buildup—exerted a more pronounced influence on deformation resistance than moderate variations in fiber volume fraction.

The comparison between different laminate architectures further reinforces this interpretation. Although the inclusion of glass fiber mat layers contributes to improved stress redistribution and damage tolerance, its effect on stiffness is secondary when compared to the dominant role of section geometry. This explains why tiles with similar fiberglass contents exhibited markedly different deformation resistance performances: the governing parameter was not reinforcement fraction alone, but the combined effect of thickness and sandwich integrity.

From a structural design standpoint, these correlations demonstrate that the efficiency of the developed roofing tiles arises from the synergy between an ultralight lignocellulosic core and fiberglass-reinforced polymer faces. Rather than relying on increased material consumption or higher reinforcement content, the proposed system achieves enhanced stiffness through optimized geometry. This finding is particularly relevant for roofing applications, where reduced self-weight, controlled deflections, and compliance with serviceability criteria are critical performance requirements.

Overall, the cross-correlative analysis confirms that the miriti-based sandwich configuration is not merely a sustainable alternative material solution, but a structurally efficient design strategy. By leveraging the low density of the core to maximize thickness and moment of inertia, the composite tiles achieve high stiffness-to-weight ratios, validating the engineering rationale behind the adopted architecture and reinforcing the potential of miriti wood for advanced lightweight structural applications.

### 3.7. Statistical Analysis

#### 3.7.1. Descriptive Statistical Analysis of Physical and Mechanical Parameters

Table 10 summarizes the main descriptive statistical indicators associated with the physical properties of the miriti core and the global mechanical response of the composite roofing tiles.

The miriti wood exhibited an extremely low mean density, confirming its suitability as an ultralight sandwich core. The coefficient of variation of approximately 8.8% reflects the intrinsic heterogeneity of lignocellulosic materials and is consistent with values reported in the literature. Despite this variability at the material level, the dispersion did not translate into loss of structural integrity at the component level.

The apparent flexural properties showed moderate to high coefficients of variation, particularly for the flexural modulus. This dispersion is attributed primarily to geometric variability, local thickness differences, impregnation quality, and the presence of defects inherent to manual lay-up processes, rather than to differences in fiber content or core density alone.

#### 3.7.2. Statistical Interpretation of Water Uptake Behavior

The water absorption results exhibited a clear time-dependent increase from 2 h to 24 h immersion, accompanied by a significant increase in dispersion, as shown in Table 10. The wider range observed after 24 h indicates that long-term moisture uptake is governed by microstructural characteristics such as porosity distribution, vessel connectivity, and local density variations in the miriti core.

From a statistical standpoint, this increase in variability is expected for lignocellulosic materials and does not, by itself, imply structural unreliability. Importantly, no degradation of mechanical performance or normative compliance was observed, indicating that the encapsulated sandwich configuration effectively decouples moisture-related variability from structural behavior.

#### 3.7.3. Exploratory Correlation Analysis Between Geometry and Structural Performance

To identify the dominant parameters governing deformation resistance, exploratory Pearson correlation coefficients were calculated between key structural variables. The results are summarized in Table 11.

The correlation analysis was performed using one data point per roofing tile (*n* = 5), considering average geometric and physical parameters and the corresponding admissible load at the serviceability limit (*L*/40). The reported Pearson coefficients are intended for exploratory interpretation only, without inferential statistical testing.

The very strong trend between total thickness and admissible load confirms that deformation resistance is predominantly governed by geometric effects, particularly the increase in second moment of area associated with thicker sandwich sections. Conversely, the weak correlation between core density and admissible load demonstrates that variations in miriti density play only a secondary role in the global structural response.

These findings are fully consistent with classical sandwich mechanics, in which the core acts primarily as a geometric spacer and shear-transfer medium, while the faces carry the dominant tensile and compressive stresses.

Although miriti wood exhibits an extremely low mean specific gravity (0.091 g/cm^3^) with a coefficient of variation of 8.8%, its quantitative influence on the structural response of the sandwich tiles was found to be secondary when compared to geometric parameters. Exploratory correlation analysis revealed a very weak linear relationship between miriti core density and deformation resistance at the serviceability limit (Pearson *r* = 0.18, *n* = 5), indicating that variations in core density within the measured range do not significantly affect the admissible load required to reach the *L*/40 deflection criterion.

Similarly, no consistent trend was observed between miriti density and apparent flexural stress or apparent flexural modulus obtained from ASTM D790 [18] tests, whose dispersion was primarily governed by geometric variability, laminate architecture, and local thickness differences rather than by core density. These results quantitatively confirm that, within the investigated density range, the miriti core acts predominantly as a geometric spacer and shear-transfer medium, while deformation resistance and apparent flexural response are dominated by sandwich geometry and face laminate integrity rather than by intrinsic core density.

#### 3.7.4. Mass-Normalized Structural Efficiency

To assess stiffness-to-weight efficiency, the admissible load values were normalized by the total mass of each roofing tile. The resulting mass-normalized performance indicators are presented in Table 12.

The results indicate significant variation in efficiency among the tested tiles, with load-to-mass ratios ranging from approximately 15.5 to 27.2 kg/kg. Although Tile 01 supported the highest absolute load (104.3 kg), its higher mass (4.28 kg) resulted in a moderate efficiency value (24.3 kg/kg). In contrast, Tile 05 exhibited the highest load-to-mass ratio (27.2 kg/kg), despite sustaining a lower absolute load (64.3 kg), due to its substantially reduced mass (2.37 kg). This behavior underscores that structural efficiency is governed not only by maximum supported load, but primarily by the interaction between geometry, mass distribution, and sandwich architecture.

Tiles with lower efficiency values, such as Tile 03 (15.5 kg/kg), combined reduced thickness with limited stiffness, reinforcing the dominant role of total section thickness in controlling deformation resistance. These findings confirm that geometric effects, particularly those related to the second moment of area, are the main drivers of structural performance, while material density plays a secondary role when an appropriate sandwich configuration is employed.

Overall, the analysis demonstrates that miriti-based sandwich tiles can achieve high stiffness-to-weight efficiency, comparable or superior to conventional roofing solutions reported in the literature. The load-to-mass ratio emerges as a robust and meaningful metric for assessing lightweight composite roofing systems, reinforcing the suitability of miriti wood as a sustainable core material when structural performance is evaluated from an efficiency-oriented engineering perspective.

#### 3.7.5. Normative Statistical Performance and Reliability

From a conformity-based statistical perspective, all tested roofing tiles met the requirements of ABNT NBR 16753 [14] with respect to deformation resistance, impact performance, and visual integrity. The approval rate was therefore 100%, indicating a high level of reliability and robustness.

This result is particularly significant given the variability observed in material properties and geometry. Rather than minimizing variability at the material level, the adopted sandwich architecture accommodates it structurally, ensuring consistent serviceability performance and compliance with safety criteria.

#### 3.7.6. Statistical Implications for Structural Design

Overall, the statistical analysis demonstrates that the performance of the miriti-based composite roofing tiles is governed primarily by geometric design and sandwich mechanics, rather than by isolated material properties. The integration of descriptive statistics, exploratory correlations, mass-normalized metrics, and conformity-based assessment provides a comprehensive and engineering-oriented interpretation of the experimental results.

These findings confirm that miriti wood, despite its intrinsic variability, can be reliably employed as a sustainable core material in sandwich composite roofing systems when appropriate geometric design principles and encapsulation strategies are applied.

### 3.8. Impact Test

Table 13 summarizes the impact resistance performance of the composite tiles according to ABNT NBR 16753 [14]. Individual specimen-level data were condensed to highlight the overall structural response and compliance with the standard.

The results presented in Table 13 show that all the composite roof tiles developed, regardless of the architecture (Type I or Type II), fully met the impact resistance requirements established by ABNT NBR 16753 [14], being approved at all performance levels evaluated, from the minimum level to the severe level of stress. In none of the 50 test specimens was rupture, penetration or permanent deformation of the material observed, which demonstrates the high capacity of the miriti/fiberglass composite system to withstand concentrated impact stresses.

Qualitative analysis of the recorded occurrences indicates that the observed damage was mostly limited to light marks on the gel coat, and, in a few cases, to more evident superficial damage also confined to the outer coating, without compromising the structural integrity of the laminate or the miriti wood core. This behavior is particularly relevant from a functional point of view, since the standard allows for superficial failures at the most severe impact levels, provided there is no loss of structural capacity or penetration of the test specimen. Thus, even in cases where the gel coat showed visible marks, the overall performance of the tile remained satisfactory.

For practical contextualization, commercially available composite roofing systems based on glass fiber reinforced polymers are typically required to withstand impact energy levels on the order of 2–5 J for service-related actions, such as accidental tool drops, localized foot traffic, or hail events, depending on laminate thickness, architecture, and manufacturer specifications. Higher impact energies, typically in the range of 5–10 J, are commonly adopted in qualification or safety-oriented assessments to ensure resistance against severe localized impacts.

In this context, the full approval of the miriti-based sandwich tiles at impact energy levels between 2 and 8 J indicates that their impact performance is consistent with, and in several cases exceeds, the lower and intermediate impact resistance thresholds reported for commercially available composite roofing systems.

From a mechanical standpoint, this result can be attributed to the synergy between the miriti core and the fiberglass faces, a typical characteristic of sandwich-type structures. Miriti wood, known for its low density and high deformation capacity, acts as an energy-dissipating element, absorbing a significant portion of the impact through local deformation mechanisms and stress redistribution. Simultaneously, the outer fiberglass layers and the polyester matrix provide sufficient rigidity to prevent perforation and localized collapse, even under the highest energy levels applied in the test.

Comparing the two evaluated architectures, it is observed that both presented equivalent performance in terms of regulatory approval. Based on the adopted criteria, it is not possible to identify a clear superiority of one configuration over the other regarding impact resistance. This equivalence suggests that, for the analyzed energy range, the impact absorption mechanism is dominated by the core-laminate assembly, and not exclusively by the sequence of fiberglass layers. However, the occasional occurrence of more pronounced marks in the gel coat in some samples indicates that local variations in thickness, impregnation, or surface finish may influence the visual impact response, even if they do not affect structural performance.

In critical terms, it is important to highlight that the normative impact test is essentially qualitative/approval-based, not providing quantitative values of absorbed energy or internal damage. Thus, although the results in Table 13 unequivocally confirm the adequacy of the tiles to the normative requirements, complementary studies—such as instrumented impact tests, internal damage analysis through detailed visual inspection, or non-destructive techniques—could provide a deeper understanding of the energy dissipation mechanisms and the influence of the laminate architecture.

### 3.9. Integrated Structural Interpretation and Scientific Implications

Beyond individual test outcomes, the combined experimental results reveal a coherent structural behavior pattern governed primarily by sandwich mechanics rather than isolated material properties. The cross-analysis between density, total thickness, deformation resistance, flexural response, and mass-normalized efficiency demonstrates that geometric amplification of stiffness plays a dominant role in structural performance.

The strong correlation between total thickness and admissible load (r = 0.93) confirms that the second moment of area effect, inherent to sandwich configurations, overrides moderate variability in core density. Conversely, the negligible correlation between miriti density and deformation resistance quantitatively reinforces that the core functions mainly as a geometric spacer and shear-transfer medium, not as a primary load-bearing element.

The flexural stress–strain curves further highlight distinct damage evolution mechanisms between laminate architectures. Type I configurations exhibited extended post-peak deformation capacity, indicating progressive damage and energy dissipation, while Type II configurations demonstrated higher initial stiffness with comparatively more brittle response. These differences illustrate how laminate architecture influences damage tolerance without altering the fundamental geometry-driven stiffness mechanism.

When moisture uptake results are interpreted in conjunction with mechanical performance, it becomes evident that encapsulation effectively decouples hygroscopic variability from structural reliability. Although long-term immersion increases statistical dispersion in water absorption, no corresponding degradation in deformation resistance or impact compliance was observed, reinforcing the robustness of the sandwich system under the evaluated conditions.

From a scientific standpoint, these findings contribute to the broader understanding of sustainable sandwich composites by demonstrating that ultralight natural cores can achieve normative structural performance when geometry-driven design principles are properly applied. Rather than focusing solely on intrinsic material properties, the present study emphasizes structural configuration as the governing parameter for component-scale performance.

## 4. Conclusions

This study demonstrated that miriti-based sandwich composite roofing tiles can achieve high structural efficiency and full normative compliance when properly designed according to geometry-driven sandwich principles. Despite the intrinsic heterogeneity and hygroscopic nature of miriti wood, its ultra-low density enables a significant increase in section thickness without penalizing mass, resulting in enhanced stiffness through second moment of area effects.

Experimental evaluation according to ABNT NBR 16753 confirmed that all manufactured tiles met the mandatory serviceability, impact resistance, and visual integrity requirements, achieving a 100% approval rate. Deformation resistance and mass-normalized performance analyses showed that structural behavior is governed primarily by geometry and laminate integrity, while variations in core density play a secondary role.

Water absorption tests on sealed sandwich specimens revealed moderate system-level moisture uptake, which did not compromise mechanical integrity or normative performance, highlighting the effectiveness of encapsulation strategies in mitigating moisture-related concerns associated with lignocellulosic cores.

Overall, the results confirm that miriti wood can be reliably employed as a sustainable and lightweight core material in sandwich composite roofing systems. The proposed design concept combines structural efficiency, variability tolerance, and normative robustness, offering a viable and environmentally responsible alternative to conventional roofing solutions. Future studies addressing long-term durability, cyclic environmental conditioning, and post-aging mechanical characterization are recommended to further consolidate the applicability of this approach and to quantify long-term performance retention. Future studies addressing long-term environmental conditioning, including cyclic wet–dry exposure and durability assessment, are recommended to further establish the long-term performance envelope of miriti-based sandwich roofing systems.

## Figures and Tables

**Table 1:** Composition of the gel coat used.

Material	Percentage Relative to the Reference	Mass (g)
Polyester Resin	Reference	400.00
Industrial Talc	15.00	60.00
Titanium Dioxide (TiO_2_)	10.00	40.00
Aerosil	3.50	14.00
Methyl Ethyl Ketone Peroxide (MEK-P)	1.00	4.00
**Total Mass (g)**		**518.00**

**Table 2:** Materials used in the manufacture of roof tiles and their respective masses.

Tile	Matrix (g)	Fiberglass (g)		MEK-P (g)	Miriti (g)	Gel Coat (g)	Tile Mass (g)
		Fabric	Blanket				
Tile 1	2195.00	309.00	873.00	21.80	366.50	518.50	4283.80
Tile 2	2414.80	390.40	910.00	23.90	414.00	518.50	4671.60
Tile 3	1071.00	577.00	-	10.40	381.00	518.50	2557.90
Tile 4	1203.70	648.20	-	11.80	491.10	518.50	2873.30
Tile 5	1099.00	593.00	-	10.90	144.50	518.50	2365.90

**Table 3:** Classification and geometric characteristics of the manufactured roofing tiles according to ABNT NBR 16753 [14].

Architecture	Sample	Laminated Type	Protection Rating	Width (mm)	Length (mm)	Total Thickness (mm)	Mass per Unit Area (kg/m^2^)
Type I	Tile 1	Opaque	Grade 1	600	1410.00	11.82	4.50
	Tile 2	Opaque	Grade 1	600	1410.00	14.56	4.08
Type II	Tile 3	Opaque	Grade 1	600	1405.00	9.74	2.37
	Tile 4	Opaque	Grade 1	610	1410.00	10.28	2.26
	Tile 5	Opaque	Grade 1	630	1390.00	12.95	2.30
**Averages**		**-**	**-**	**608**	**1405.00**	**11.87**	**3.10**

**Table 4:** Geometric dimensions adopted in the different experimental tests.

Element	Dimension	Description
Total tile thickness	9.7–14.6 mm	Overall thickness of the sandwich roofing tiles, varying with architecture and laminate build-up
Local coupon thickness (flexural test)	≈12 mm	Average thickness measured in the flat region from which flexural specimens were extracted
Water absorption specimen dimensions	25 mm × 25 mm × 20 mm	The 20 mm dimension corresponds to the full sandwich height, ensuring inclusion of faces and miriti core

**Table 5:** Fiberglass content incorporated into each manufactured tile.

Architecture	Test Specimens (Tiles)	Fiberglass Content
Type I	Tile 1	31.39%
	Tile 2	31.31%
Type II	Tile 3	28.29%
	Tile 4	27.53%
	Tile 5	32.10%

**Table 6:** Specific gravity of miriti wood determined according to ABNT NBR 7190 [16]–based procedure.

Number of Samples	Mean Specific Gravity (g/cm^3^)	Standard Deviation (g/cm^3^)	Coefficient of Variation (%)	Minimum (g/cm^3^)	Maximum (g/cm^3^)
10	0.091	0.008	8.8	0.078	0.105

**Table 7:** Apparent flexural properties obtained from ASTM D790 [18] three-point flexural tests.

Architecture	Sample	Apparent Flexural Stress (MPa)	Apparent Flexural Strain (–)	Apparent Modulus of Elasticity (GPa)
Type I	Tile 1	6.72 ± 1.65	0.028 ± 0.004	0.707 ± 0.168
Type II	Tile 3	9.27 ± 2.85	0.012 ± 0.003	1.877 ± 0.943
	Tile 4	7.20 ± 1.80	0.014 ± 0.003	1.454 ± 0.296
	Tile 5	6.75 ± 0.44	0.011 ± 0.002	1.177 ± 0.178

**Table 8:** Applied mass and equivalent force required to reach the admissible deflection limit (*L*/40).

Tile	Deflection Limit (mm)	Applied Mass (kg)	Equivalent Force (N)
Tile 1	15.0	104.3	1023
Tile 2	15.0	92.0	903
Tile 3	15.0	39.6	388
Tile 4	15.25	49.2	483
Tile 5	15.75	64.3	631

Note: The admissible deflection limits were calculated using the effective clear span between supports (≈600–630 mm), in accordance with ABNT NBR 16753 [14], and not the total length of the loading platform.

**Table 9:** Comparative flexural performance with literature data.

Tile System	Maximum Load (kg)	Deflection (mm)	Failure
Cementitious tile (0% residue) [21]	60	37	Yes
Cementitious tile (50% residue) [21]	85	48	Yes
Miriti composite tile (this work)	104.3	15	No

**Table 10:** Descriptive statistics of physical and structural parameters of the composite roofing tiles.

Parameter	Mean	Std. Dev.	CV (%)	Min	Max
Miriti core density (g/cm^3^)	0.091	0.008	8.8	0.078	0.105
Fiberglass content (%)	≈30	—	—	27.5	32.1
Water uptake–2 h (%)	≈2.5	—	—	≈0	≈5
Water uptake–24 h (%)	≈6.0	—	—	≈1	≈11
Apparent flexural stress (MPa)	6.7–9.3	0.4–2.8	6–30	—	—
Apparent flexural modulus (GPa)	0.7–1.9	0.17–0.94	15–50	—	—

**Table 11:** Pearson correlation coefficients between geometric parameters and deformation resistance of the roofing tiles.

Correlated Variables	Pearson *r*	*n*	Data Points Used	Interpretation
Total tile thickness × admissible load (*L*/40)	0.93	5	Tiles 1–5	Very strong trend
Mass per unit area × admissible load (*L*/40)	0.71	5	Tiles 1–5	Moderate positive correlation
Miriti core density × admissible load (*L*/40)	0.18	5	Tiles 1–5	Weak/negligible correlation

**Table 12:** Mass-normalized deformation resistance of the composite roofing tiles.

Tile	Tile Mass (kg)	Supported Load (kg)	Load-to-Mass Ratio (kg/kg)
Tile 1	4.284	104.3	24.34
Tile 2	4.672	92.0	19.70
Tile 3	2.558	39.6	15.49
Tile 4	2.873	49.2	17.13
Tile 5	2.366	64.3	27.17

**Table 13:** Summary of impact resistance performance.

Tile Architecture	Number of Specimens	Impact Energy Levels (J)	Structural Failure	Observed Damage
Type I	20	2.0, 3.7, 5.0, 8.0	No	Superficial marks on gel coat
Type II	30	2.0, 3.7, 5.0, 8.0	No	No structural damage observed

Note: Impact energies were varied by changing the impactor mass while maintaining a constant drop height of 1.0 m.

## Data Availability

The original contributions presented in the study are included in the article; further inquiries can be directed to the corresponding author.
